# Mott state of flat bands in a 2D metal–organic Kagome framework

**DOI:** 10.1093/nsr/nwaf574

**Published:** 2025-12-15

**Authors:** Tianchen Qin, Xingyue Wang, Jia Wang, Xiaoyin Li, Sifan You, Zihan Wang, Yiyang Yin, Lizhi Zhang, Junfa Zhu, Feng Liu, Lifeng Chi, Minghu Pan

**Affiliations:** National Synchrotron Radiation Laboratory, Department of Chemical Physics and State Key Laboratory of Precision and Intelligent Chemistry, University of Science and Technology of China, Hefei 230029, China; School of Physics and Information Technology, Shaanxi Normal University, Xi’an 710119, China; School of Physics and Information Technology, Shaanxi Normal University, Xi’an 710119, China; Department of Materials Science and Engineering, University of Utah, Salt Lake City, UT 84112, USA; State Key Laboratory of Bioinspired Interfacial Materials Science, Institute of Functional Nano & Soft Materials (FUNSOM), Soochow University, Suzhou 215123, China; School of Physics and Information Technology, Shaanxi Normal University, Xi’an 710119, China; Laboratory of Theoretical and Computational Nanoscience, National Center for Nanoscience and Technology, Beijing 100190, China; Laboratory of Theoretical and Computational Nanoscience, National Center for Nanoscience and Technology, Beijing 100190, China; National Synchrotron Radiation Laboratory, Department of Chemical Physics and State Key Laboratory of Precision and Intelligent Chemistry, University of Science and Technology of China, Hefei 230029, China; Department of Materials Science and Engineering, University of Utah, Salt Lake City, UT 84112, USA; State Key Laboratory of Bioinspired Interfacial Materials Science, Institute of Functional Nano & Soft Materials (FUNSOM), Soochow University, Suzhou 215123, China; School of Physics and Information Technology, Shaanxi Normal University, Xi’an 710119, China

**Keywords:** metal–organic framework, Kagome lattice, flat bands, correlated Mott states

## Abstract

Mott states of flat bands (FBs), as a representative strong correlation effect, have recently attracted much attention. However, observation of Mott states has been mostly confined to two-dimensional (2D) inorganic materials. Here, we report the observation of Mott states associated with FBs in a 2D organic quantum material. We have synthesized a large-scale uniform 2D Ag–(BPhen)_3_ metal–organic framework (MOF) of a Kagome lattice on the Ag(111) surface. Scanning tunneling microscopy/spectroscopy measurements show high density of states around the charge neutrality point within the MOF, which is consistent with density functional theory calculations that predict an FB bundle (consisting of an FB and flattened Dirac bands) located at the Fermi level (*E*_F_). Interestingly, the FB at the *E*_F_ is observed to split into upper/lower Hubbard bands (UHBs/LHBs) with a Mott insulating gap of ∼85–103 meV, as evidenced by the inverted contrast of d*I*/d*V* maps between the LHB and UHB. Furthermore, temperature-dependent measurements show a Mott transition temperature of ∼15 K, at which the Mott gap closes. By deposition of K atoms, the gap size is seen to be reduced in the electron-doped framework, which indicates the filling of the correlated Mott states distinguishable from a trivial band insulator. Also, an insulator-to-metal transition was observed approaching a K-doped defect site. Our studies demonstrate a proof of concept for correlated Mott states of topological FBs in artificially synthesized 2D MOFs, opening a new avenue to organic FB superconductivity and exotic quantum many-body phenomena in organic systems.

## Teaser

This work report the observation of Mott states associated with flat bands in a large-scale uniform 2D Kagome metal-organic framework synthesized on Ag(111) surface.

## INTRODUCTION

Two-dimensional (2D) Mott insulators emerge in strongly correlated material Kagome framework systems that host highly localized electronic states where the Coulomb interaction (described by the parameter *U*) exceeds the bandwidth (*W*) [1]. It is often linked and/or competing with other strongly correlated electronic behavior [2,3], such as high-temperature superconductivity in transition metal oxides [4,5], charge density wave (CDW) states in layered transition metal dichalcogenides [6–9] and unconventional superconductivity in multilayer graphene [10–13] and twisted bilayer graphene (TBG) [10]. However, observation of Mott states has been mostly confined to 2D inorganic materials. This is because the localized electronic states are associated with atomic *d*- and *f*-orbitals, which commonly exist in inorganic materials containing *d*- and *f*-elements, or flat bands (FBs) composed of *s*- and *p*-orbitals as exemplified in TBG.

On the other hand, the FBs can arise from lattice symmetry in certain classes of 2D lattices [14,15], which can be readily realized in various organic frameworks [16–18]. Such FBs arise from destructive quantum interference enforced by lattice symmetry, which renders the FB topological in both real space, characterized by a compact localized state, and momentum space having a singular touching point with a dispersive band [19]. It will be interesting and desirable to show the generality of Mott physics associated with the topological FBs in organic materials beyond the inorganic materials that have been studied mostly so far. Especially, the Mott physics in organic materials of metal–organic frameworks (MOFs) and covalent organic frameworks (COFs) may give rise to different manifestations without the localized atomic *d*- and *f*-orbitals. Furthermore, the electronic structures of MOFs/COFs are highly tunable through changing ligands and linkers, to facilitate deeper comparative studies. Therefore, organic frameworks provide a fertile playground to study Mott and other correlated FB physics, which remain to be more extensively explored [17].

To realize Mott-like physics in a 2D organic framework, one needs to first overcome the challenge in synthesizing a large-scale organic crystalline lattice, hosting cleanly an FB at the charge neutrality point [14]. Many 2D MOFs [18–22] and COFs, [23–25] as well as inorganic 2D materials [26], have been predicted from first principles to host FBs. However, in most systems, FBs inevitably overlap with other dispersive bands and/or are not truly flat throughout the whole Brillouin zone (BZ) [27]. For example, recently, Pan *et al.* reported the growth of a self-assembled monolayer of 2D hydrogen-bond organic frameworks (HOFs) of 1,3,5-tris(4-hydroxyphenyl) benzene (THPB) on Au(111) [28]. A clean flat band with a bandwidth less than 0.1 eV is clearly resolved by angle-resolved photoemission spectroscopy (ARPES), which indicates an effective ‘breathing’ Kagome lattice. But the FB in this system is located at an energy of −2.6 eV, far below the Fermi level (*E*_F_), which prevents the observation of FB-related correlated electronic behavior.

In this work, we successfully synthesized a nearly perfect Kagome lattice based on a metal–organic coordination Ag–(BPhen)_3_ framework (a MOF). Using *in situ* high-resolution scanning tunneling microscopy/spectroscopy (STM/STS), we observed a series of density of states (DOS) peaks within the Ag–(BPhen)_3_ Kagome framework, consistent with density functional theory (DFT) calculations that predict a series of non-dispersive bands with small bandwidth in a broad range of energies. Especially, a distinct peak is seen at the *E*_F_, which is resolved to arise from an FB bundle [including an FB and several flattened Dirac bands (DBs)] by DFT calculations. Most remarkably, high-resolution STS spectra reveal a gap of ∼85–103 meV at the *E*_F_, while differential conductance maps taken at the two edges of the gap display inverted contrast, indicative of lower Hubbard band (LHB) and upper Hubbard band (UHB) of a Mott gap. Furthermore, temperature-dependent d*I*/d*V* spectroscopy shows fast decay of the Mott gap with increasing temperature and a gap-closure at 15 K, consistent with the fact that the Mott state, induced by an electronic correlation effect, is strongly temperature-dependent. In contrast, the DOS peaks corresponding to FBs as parts of the band structure are largely independent of temperature. Upon K doping, the d*I*/d*V* spectra measured near a K-induced defect site display a shift in the *E*_F_ and the filling of the gap, indicating the electron-doped Mott behavior. Our results demonstrate strong evidence of correlated Mott physics induced by FBs in a 2D organic MOF with mesoscale order, paving the way for future studies of MOFs as a unique class of organic quantum materials.

## RESULTS AND DISCUSSION

### Experimental synthesis and spectroscopy of FBs in a large-scale 2D Ag-(BPhen)_3_ Kagome lattice

**Figure 1.**
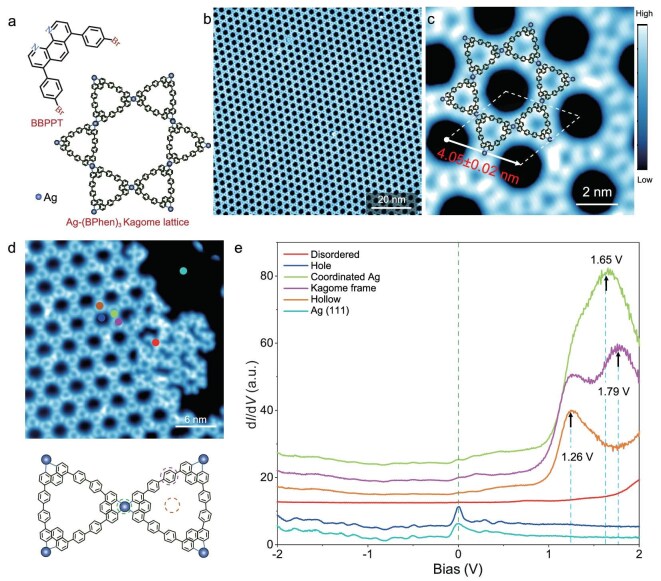
STM images and tunneling spectra of the Ag–(BPhen)_3_ Kagome lattice. (a) The fabrication of 4-fold N–Ag coordinated Kagome lattices on Ag(111) from BBPPT precursor to the Ag–BPhen_3_ Kagome structure. (b) STM image showing the large-scale uniformity of the Ag–(BPhen)_3_ Kagome lattice; the image size is 100 × 100 nm^2^ with *V*_B_ = −1000 mV and *I*_T_ = 100 pA. (c) High-resolution STM image showing the unit cell and its size of 4.05 ± 0.02 nm. The image size is 10 × 10 nm^2^ with *V*_B_ = +2200 mV and *I*_T_ = 300 pA. The structure of the Ag–(BPhen)_3_ lattice is overlaid with the image to guide the eye. (d) Upper: STM image taken near the edge of the Ag–(BPhen)_3_ Kagome lattice, where both the Kagome lattice and the Ag(111) clean surface can be visualized. The colored spots indicate the point locations where the STS was measured. The image size is 30 × 30 nm^2^ with a *V*_B_ of −1000 mV and an *I*_T_ of 100 pA. Lower: a schematic of the molecular structure marked with colored circles indicating the positions where the STS is taken. (e) Point spectra measured at the clean Ag(111) surface (cyan), disordered molecules (red), holes (blue), coordinated Ag (green), benzene bone (purple) and hollow site (orange) of the Kagome lattice. The d*I*/d*V* spectra were measured with *V*_B_ = −2000 mV, *I*_T_ = 1000 pA and a bias modulation of 25 mV at 9.2 K.

Figure 1a shows the surface-assisted synthesis of 4-fold N–Ag coordinated Kagome lattices by deposition of N-doped 4,7-bis(4-bromophenyl)-1,10-phenanthroline (BBPPT) precursors on Ag(111) substrates (see [29] for details). The 2D MOF consists of 4-fold N–Ag coordination bonds connecting the covalent trimer of bathophenanthroline (BPhen). The metal–organic coordination not only has the advantages of selectivity, directionality and reversibility, but also provides an intermolecular channel for charge transfer, as well as for strengthening Kagome lattices [30–33]. Figure 1b shows the uniformity of the Ag–(BPhen)_3_ lattice in an area of 100 × 100 nm^2^ (see Fig. S1 for larger-scale uniformity of Ag–(BPhen)_3_ lattices up to 400 × 400 nm^2^). From the high-resolution STM image in Fig. 1c, we measure the size of the unit cell of the Ag–(BPhen)_3_ lattice as ∼4.05 ± 0.02 nm [25], consisting of three coordinated Ag nodes and six BPhen bones. A comparison of high-resolution images between the Ag–(BPhen)₃ Kagome lattice and the substrate lattice reveals that the coordinated Ag adatoms (highlighted by red circles) are located at the hollow sites of the Ag(111) surface (Fig. S17). Large-scale STM images (Fig. S18) show that the MOF lattice maintains the same orientation across the steps, and identical orientations are observed on different steps.

**Figure 2.**
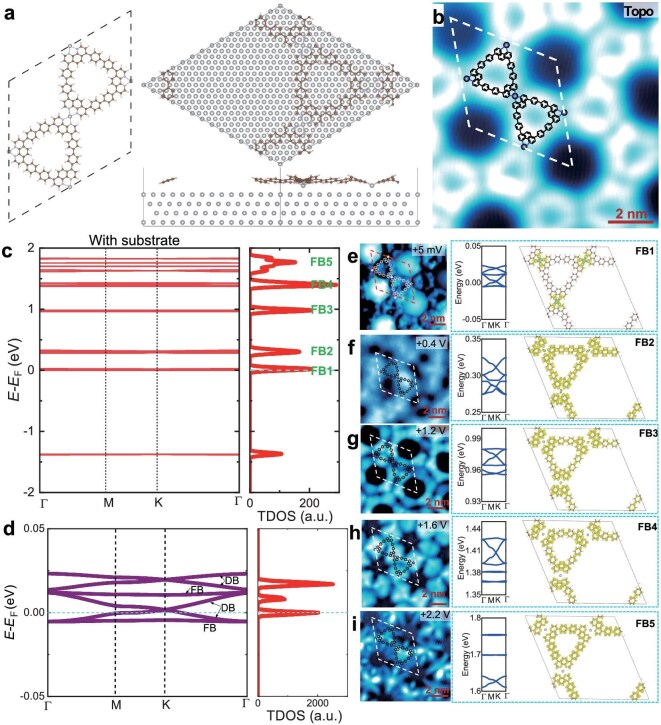
The calculated bands and spectroscopic imaging of a series of FBs in the Ag–(BPhen)_3_ Kagome lattice. (a) The optimized unit cell of the freestanding 4-fold N–Ag-coordinated Ag–(BPhen)_3_ lattice (left) and on the Ag(111) substrate modeled with four Ag layers (right). (b) High-resolution topographic image, measured at *V*_B_ = +1400 mV and *I*_T_ = 300 pA. (c) The calculated band structure of MOF on Ag(111) substrate in a large energy range from −2 eV to 2 eV, showing a series of FB bundles, FB1, FB2, FB3, FB4 and FB5 at the energies of 0, +0.3, +0.98, +1.39 and +1.77 eV, respectively. Right: the integrated DOS. (d) The zoom-in view of FBs and Dirac bands (DBs) near *E*_F_. (Right) The integrated density of states (DOS). (e–i) (Left) Spectroscopic (d*I*/d*V*) images taken at biases of +5 mV, 0.4 V, +1.2 V, +1.6 V and +2.2 V, respectively. (Right) Calculated LDOS maps for the FBs and DBs, as well as detailed band structure (middle panels). The d*I*/d*V* maps were measured with an *I*_T_ of 500 pA and a bias modulation of 25 mV at 9.2 K.

**Figure 3.**
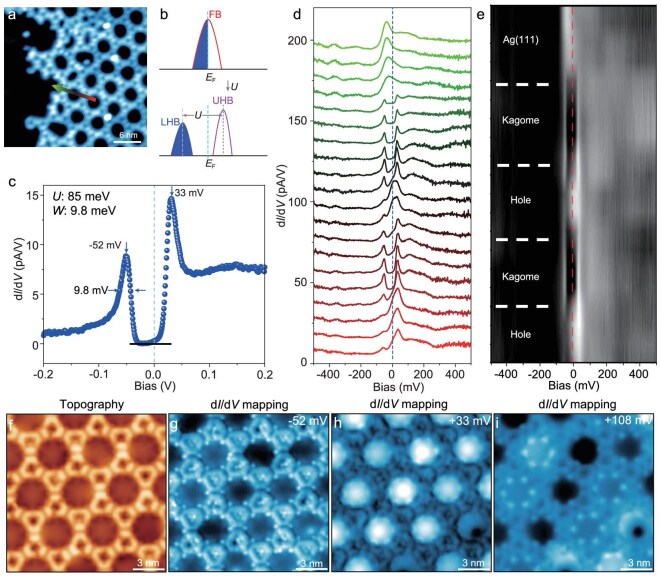
Spectroscopic signature of the Mott gap formed by the FB at the *E*_F_, measured across the Ag–(BPhen)_3_ Kagome lattice. (a) An STM image taken near the edge of the Ag–(BPhen)_3_ Kagome lattice. The measurements are at *V*_B_ = −2200 mV and *I*_T_ = 100 pA. (b) Schematics showing an FB at the *E*_F_ turning to the UHB and LHB with a Mott insulating gap. (c) A point STS measured on the Kagome lattice showing the formation of a UHB/LHB and a Mott gap of approximately 85 meV at the *E*_F_. The horizontal black bar indicates the level of zero differential conductance. (d) A line spectroscopic survey taken along the arrowed line in panel (a). All d*I*/d*V* spectra were measured with *V*_B_ = −500 mV, *I*_T_ = 1000 pA and a bias modulation of 5 mV at 2 K. (e) A gray-scale plot of site-dependent d*I*/d*V* spectra. From the top of the panel, the LDOS varies from the Ag(111) surface to the Kagome lattice, in which the UHB/LHB bands only appear on the Ag–(BPhen)_3_ Kagome lattice with a well-defined energy gap. (f) Topography and a series of d*I*/d*V* maps (g–i) taken at energies of −52 mV (LHB), +33 mV (UHB) and +108 mV, respectively. The d*I*/d*V* maps were measured with an *I*_T_ of 500 pA and a bias modulation of 5 mV at 2 K.

To identify the distinguished electronic states of this Kagome MOF lattice, we selected a region near the edge of the MOF island for imaging comparison, as shown in Fig. 1d. Point d*I*/d*V* spectra were collected at various spatial locations, e.g. the clean Ag(111) surface (cyan), disordered molecules (red), hole (blue), coordinated Ag site (green), benzene bone (purple) and hollow site (orange) of the Kagome lattice, as shown in Fig. 1e. The tunneling spectrum (cyan curve) measured on the Ag(111) surface shows representative peaks at approximately −15 mV, indicating the typical Ag(111) surface state (SS) [34], consistent with the minimum value of dispersive Shockley SS that is approximately −63 meV for the Ag(111) surface. The d*I*/d*V* spectrum measured for disordered molecules (red curve) shows no observable DOS features in the energy range of −2 to +2 eV, indicating that there are no molecular orbital states in this energy range. In contrast, the d*I*/d*V* spectra measured for the coordinated Ag site (green curve), benzene site (purple curve) and hollow site (orange curve) within the Kagome MOF lattice (Fig. 1e) show strong DOS peaks at +0.005, +1.26, +1.65 and +1.79 eV, respectively (the peak of +0.005 eV is more prominent in the enlarged d*I*/d*V* spectra in Fig. 3d). These peaks are found to be consistent with the energy positions of a series of FBs predicted by DFT calculations, i.e. the FB1, FB3, FB4 and FB5 shown in Fig. 2c (see Discussion). The reason for the absence of FB2 calculated by DFT is unknown. We note that the experimentally measured peak positions can be shifted relative to the DFT predictions due to the charge transfer between the metal substrate and molecular overlayer, which is not considered in the DFT calculations [22]. The small shift we found here implies a weak interfacial charge transfer, which agrees with our ARPES measurements (see Supplementary Note 13).

### DFT-calculated band structure and spectroscopic imaging of the FBs in the Ag−(BPhen)_3_ Kagome lattice

To better understand the experimental STS data, we performed DFT calculations. The optimized MOF structure with and without Ag(111) substrate is shown in Fig. 2a. The optimized freestanding MOF structure is shown in the left panel of Fig. 2a, which has a nearly perfect Kagome lattice made of Ag atoms. The DFT optimized MOF structure is overlaid onto the high-resolution STM image (Fig. 2b), which matches almost perfectly. The effective Ag–Ag Kagome lattice hopping is mediated via the BPhen bone and N–Ag coordination bond. The calculated band structure of the Ag–(BPhen)_3_ with Ag(111) substrate is shown in Fig. 2c, exhibiting a series of FB bundles: FB1, FB2, FB3, FB4 and FB5, located at the energies of 0, +0.30, +0.98, +1.39 and+1.77 eV, respectively, in the energy range from −2 eV to +2 eV; the corresponding integrated DOSs are shown in the right panels. It can be seen that each bundle of FBs and flattened DBs will introduce a DOS peak. Most importantly, there is an FB bundle located at the *E*_F_ (Fig. 2c and d) and a prominent DOS peak at the *E*_F_ (right panel of Fig. 2d). The FB1 is located rightly at *E*_F_, which facilitates the study of Mott physics. This feature is important and useful because the FBs of the organic Kagome lattice mostly lie far from the *E*_F_ due to the random sub-band positions and band filling. Our system contains a series of FBs, which allow the *E*_F_ to coincide with one set of FBs for different band fillings without the need of doping. FB3 and FB4 could be upshifted by ∼0.26–0.28 eV, corresponding to the experimental DOS peaks at +1.26 and +1.65 eV, respectively. Also, the d*I*/d*V* spectrum measured with a closer tip-sample distance (*U*_B_ = −500 mV; *I*_T_ = 500 pA) shows a broader DOS peak at ∼0.39 eV, as shown in Fig. S22a, which might be attributed to FB2. Consequently, the DFT-predicted FB1, FB2, FB3, FB4 and FB5 positions correspond reasonably well with the experimental DOS peaks in the d*I*/d*V* curve, e.g. +0.005, +0.40, +1.26, +1.65 and +1.79 eV, respectively.

To further confirm the assignments of these FBs, we performed spectroscopic mapping (d*I*/d*V* mapping) on the Kagome MOF lattice. The simultaneously measured d*I*/d*V* maps at energies of +5 mV, +0.4 V, +1.2 V, +1.6 V and +2.2 V are shown in Fig. 2e–i. These mappings reveal the distribution of FB electrons on the Kagome lattice, which are mapped onto the calculated local DOS (LDOS) of the corresponding DFT-calculated FBs, as shown in the right panels of Fig. 2e–i. The d*I*/d*V* map at +5 mV (Fig. 2e) shows basically the brightness in the hole, corresponding to the Ag SS localized inside the hole due to the strong screening effect of Ag SS. Besides these bright holes, another feature observed in Fig. 2e is the texture of the Kagome framework, which is consistent with the calculated LDOS map of FB1 shown on the right. The d*I*/d*V* map at +0.4 V (Fig. 2f) shows a delocalized DOS distribution, in contrast to other d*I*/d*V* maps at the energies of FBs, which agree well with the broad DOS peak in the d*I*/d*V* curve in this energy range. The d*I*/d*V* map at +1.2 V (Fig. 2g) shows the brightness at locations of the benzene bone of the Kagome lattice, which agrees with the calculated LDOS map of FB4 shown on the right. The d*I*/d*V* maps at +1.6 V (Fig. 2h) and +2.2 V (Fig. 2i) correspond to the calculated LDOS maps of FB2 and FB5, respectively. In these cases, the d*I*/d*V* maps do not agree well with the calculated LDOS maps, which could be attributed to the fact that our calculations did not consider the possible effects of defects of impurities. Also, the spectroscopic measurements show that the DOS peaks at +1.26, +1.65 and +1.79 eV only exist on the Kagome lattice (see Fig. S2), indicating that these states originate from the Kagome lattice.

### Spectroscopic signature of FB-induced Mott states

A partially filled FB is unstable due to the large DOS at the *E*_F_. It has been shown that even an arbitrarily small Coulomb interaction may lead to different many-body quantum states at specific partial fillings, e.g. ferromagnetic ground state [18,35,36] and fractional quantum Hall effect [37]. In this regard, we have performed small-range d*I*/d*V* measurements with slightly modulated biases at lower temperature of 2 K, to obtain high-resolution spectra. A linear STS survey was measured along the line in Fig. 3a. It enabled us to reveal a gap-like line shape around the *E*_F_ in the d*I*/d*V* spectrum of the Ag–(BPhen)_3_ Kagome lattice (Fig. 3c). The line shapes of both the left- and right-side peaks around the *E*_F_ can be nicely fitted by the corresponding UHB and LUB of a Mott insulator, respectively, using a narrow bandwidth (*W* ∼ 9.8 meV) of the FB (blue dotted curve in Fig. 3c) and assuming a much larger correlation potential of *U* ∼ 85 meV. Another similar measurement gives the correlation potential of *U* ∼ 103 meV (Fig. S3). Compared to a previously reported Mott gap of ∼200 meV in a MOF [25], the Mott gap in this work is smaller. Furthermore, the line spectroscopy survey and its gray plot (Fig. 3d and e) confirmed that such gap-like features exist only within the Kagome MOF lattice. We note that such a gap did not appear in Fig. 1e, which might be caused by a higher measuring temperature of ∼9.2 K, and/or the lower resolution of STS (±2 eV).

One spectroscopic signature of the Mott state is the inverted contrast of the LDOS maps between the UHB and LHB [18]. Indeed, we have confirmed such a contrast by acquiring the d*I*/d*V* maps at −52 meV (the LHB position, Fig. 3g) and +33 meV (the UHB position, Fig. 3h). It can be seen that for the inverted contrast, the brighter regions in Fig. 3g are at the frames of the Kagome lattice, while the bright regions in Fig. 3h are in the holes of the Kagome lattice. This indicates that the electronic DOS of the LHB is located within the frame of the Kagome lattice, while that of the UHB is pushed into the holes of the Kagome lattice. We also present the d*I*/d*V* map at +108 mV (Fig. 3i) and topographic image (Fig. 3f) for comparison.

### Temperature dependence of the gap

**Figure 4.**
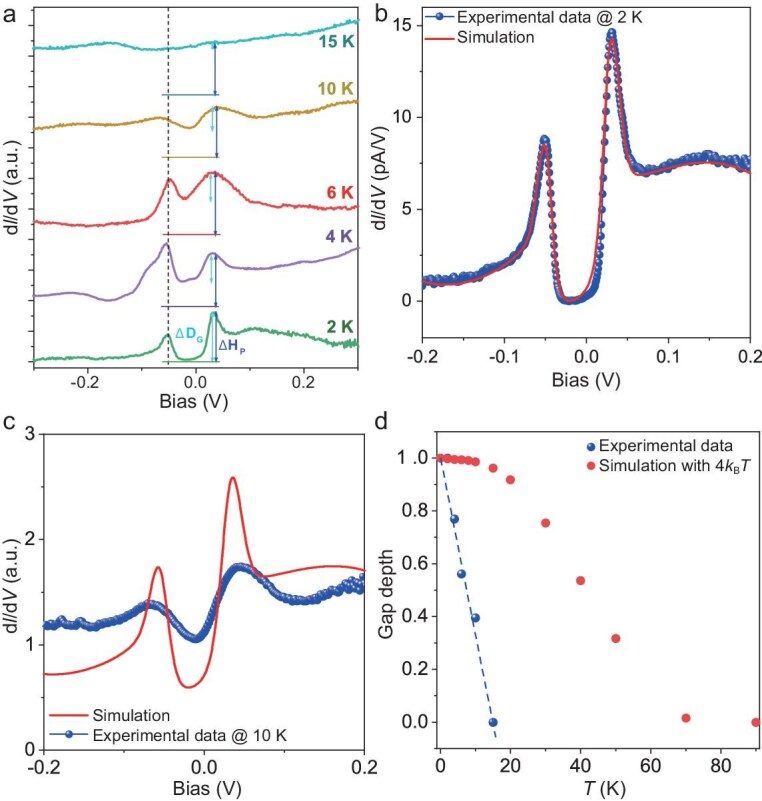
Temperature dependence of Mott gap. (a) d*I*/d*V* spectrum taken on Ag–(BPhen)_3_ Kagome measured at various temperatures, e.g. 2, 4, 6, 10 and 15 K. The horizontal bars indicate the level of zero differential conductance. The ΔD is defined by the ratio of the ΔD_G_ (cyan arrow) and the peak height (ΔH_P_, blue arrow). All d*I*/d*V* spectra were measured with *V*_B_ = −500 mV, *I*_T_ = 200 pA and a bias modulation of 8 mV. (b and c) Comparison between experimental data (blue dots) and simulation result (red curve) at 2 K (b) and 10 K (c), respectively. (d) Comparison of the ΔD between the experimental data (blue dots) and simulation results with 4*k_B_T* thermal broadening (red dots). The same procedure was used for the simulated curves in Fig. 4b and c and Fig. S10.

To further characterize the observed gap, we measured its evolution with temperature. As shown in Fig. 4a, the gap gradually becomes shallower with the increasing temperature and closes at ∼15 K. Clearly, the observed gap closure cannot be explained solely by thermal broadening. In Fig. 4b, the 2 K spectrum (blue dotted) was fitted nicely by simulating the thermal broadening at *T* = 2 K (see Supplementary Note 10 for details of simulation). The simulated 10 K data (red curve) significantly deviates from the measured 10 K spectrum (blue dotted) in Fig. 4c. To quantify the gap evolution over a wide temperature range, we define the gap depth ΔD by the ratio of ΔD (ΔD_G_, cyan arrow) and peak height (ΔH_P_, blue arrow) in Fig. 4a. Figure 4d shows the simulated ΔD and the experimentally measured value as a function of temperature; the experimental results exhibit a more rapid decay. This spectroscopic behavior indicates that the observed gap at the *E*_F_ is due to electron correlation effects [38,39], resulting in a correlated insulating state rather than a single-particle band insulator, as reported in monolayer Mo_5_Te_8_ [40] and Mo_33_Te_56_ [41]. We note that the Coulomb repulsion of *U* ∼ 85 meV in our work is smaller than the previously reported Mott gap of ∼200 meV in MOF [25], and much smaller than the *U* value in some well-known inorganic Mott insulators, such as 0.6 eV in VO_2_ [42] and 1.1 eV in Nb_3_Cl_8_ [43]. The smaller *U* leads consistently to a lower temperature of gap closing.

### Gap evolution upon potassium doping

The Mott insulating state is driven by strong on-site Coulomb interactions of electrons for a material with a partially filled narrow band, which would otherwise form a metallic state [44]. It has been shown previously that one way to possibly distinguish a Mott insulator from a trivial band insulator, as exemplified in 1T-TaS_2_, is by electron doping; the partially filled electron in a Mott insulator is fully saturated by the electron donated from K. The filled electron orbital is strongly localized, so that the surrounding lattice sites feel little doping effect. In contrast, a rigid shift upon doping is expected for a band insulator [45].

Therefore, we have doped K into the Kagome MOF structure under ultrahigh vacuum (UHV) with a base pressure lower than 1 × 10^−10^ Torr. We found that the original Ag–(BPhen)_3_ Kagome lattice will be destroyed at K coverages greater than 0.04 monolayer (ML), so the following discussions are based on a lower coverage of <0.04 ML. After K doping, the original Ag–(BPhen)_3_ Kagome lattice becomes disordered with the appearance of numerous defects (see Fig. S5). By taking high-resolution images of these defects, we found that they include an extra BBPPT molecule embedded in the pores of the original Ag–(BPhen)_3_ Kagome lattice, called the ‘BBPPT-embedded defect’. Similar defects are also occasionally found in the original Ag–(BPhen)_3_ Kagome lattice (see Fig. S4). To determine the configuration of these defects, we measured the synchrotron radiation photoemission spectroscopy (SRPES) of the K-doped samples with varying K coverages (Supplementary Note 12, Figs S12 and S13) [46]. We found that K atoms tend to form 2-fold N–K coordination bonds with N instead of 4-fold coordination bonds, disrupting the Kagome lattice structure due to the significantly larger atomic radius of K compared to Ag. Based on our SRPES results, we propose a structural model for ‘BBPPT-embedded defect’ (see Fig. S5).

**Figure 5.**
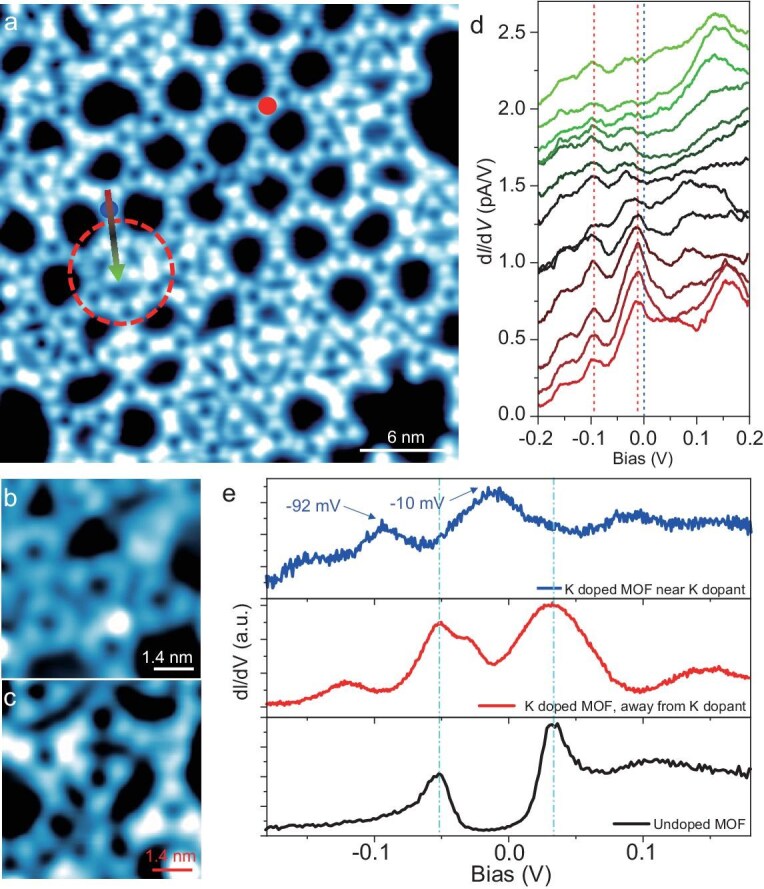
Destructive Ag–(BPhen)_3_ Kagome lattice and gap evolution after <0.04 ML K doping measured at 4 K. (a) STM image showing the destructive Ag–(BPhen)_3_ Kagome lattice after K doping. The image size is 30 × 30 nm^2^ with *V*_B_ = +300 mV and *I*_T_ = 10 pA. (b and c) Zoom-in image showing defects in the Ag–(BPhen)_3_ Kagome lattice after K doping. Both defects are Kagome units with an embedded (BPhen)_3_. The image size is 7 × 7 nm^2^ with *V*_B_ = +300 mV and *I*_T_ = 10 pA. (d) A series of d*I*/d*V* spectra measured along the colored arrow in panel (a). The Kagome lattice remains intact and adjacent to a (BPhen)_3_-embedded Kagome unit. Thus, such a d*I*/d*V* spectrum can represent the K-doped Ag–(BPhen)_3_ Kagome lattice. The d*I*/d*V* curves were measured with a *V*_B_ of +300 mV, an *I*_T_ of 10 pA and a bias modulation of 2 mV at 4 K. (e) Comparison of the d*I*/d*V* spectra between the undoped Ag–(BPhen)_3_ Kagome (black), K-doped Ag–(BPhen)_3_ Kagome lattices near the dopant (blue) and away from the dopant (red). The gap size is ∼82 meV after K doping and the left-shift is ∼40 meV, corresponding to electron doping. The gap is slightly smaller upon the filling up of DOS. The colored d*I*/d*V* curves are measured at the locations of the corresponding colored dots in panel (a).

We measured the d*I*/d*V* along the frame of the Kagome lattice, which is adjacent to a BBPPT-embedded defect, as shown in Fig. 5a. A series of d*I*/d*V* spectra in Fig. 5d show the peak–dip feature, labeled with red dashed lines. By plotting a typical d*I*/d*V* spectrum in Fig. 5e, we found that the gap size becomes ∼82 meV upon K doping, with a ∼40 meV left shift, indicative of electron doping. Thus, the K doping decreases the gap size, as well as making the gap shallower, while increasing the DOS. Most noticeably, the spectral weights of the LHB and UHB in the d*I*/d*V* spectra change greatly, with a significantly increased peak width (bandwidth). The *E*_F_ is shifted slightly toward the UHB. However, the overall characteristics of the spectra remain the same, as exemplified by the plot in Fig. 5e. This can be understood by considering that the doped electrons are trapped by defects for a small amount of K deposition. When the tip approaches a defect (brown curve), additional excitations become identifiable within the Mott gap that lie close to the UHB, indicating the electron-doped Mott insulator. When the tip is placed right on the defect, the whole Mott gap is filled up (see Fig. S6). At the defect, the collected d*I*/d*V* results exhibit a finite electronic state at the *E*_F_ on the MOF lattice. On the other hand, the STS measured at the locations away from the doping sites shows a similar Mott gap and LHB/UHB as undoped MOF. These STS results further support the assignment of a Mott gap [45]. In general, the K doping transfers electrons to the MOF lattice, so that doping will increase electron band filling. This may explain why both the UHB and LHB are shifted to lower energies, with the UHB being at −10 meV. This suggests that more than one electron is doped into the UHB ‘locally’ very close to the K dopant so that the local Mott gap becomes also much shallower than that of the undoped case (see Fig. 5e), indicating decay or removal of the Mott state by K doping locally. Away from K, the *E*_F_ is in between the LHB and UHB, although the Mott gap is less sharp, indicating the charge transfer is rather local and non-uniform.

## CONCLUSION

In summary, we fabricated large-scale uniform Ag–(BPhen)_3_ Kagome lattices on the Ag(111) surface. A series of FBs and DBs, especially one FB bundle (including an FB and several DBs) at the charge neutrality point, are observed by STM/STS measurements aided by DFT calculations. This enabled us to reveal a Mott transition at ∼15 K, with a Mott gap of 85–103 meV, as evidenced by the inverted contrast between the d*I*/d*V* maps of the UHB and LHB and signature Mott gap evolution with temperature. Also, K doping shows a shift of the *E*_F_ and filling of the Mott gap, distinguishable from the behavior of a trivial band insulator. Our results demonstrate a proof of concept for Mott states of topological FBs as hosted in a Kagome lattice in artificially synthesized 2D MOFs, facilitating future exploration of exotic quantum many-body phenomena, such as unconventional superconductivity and fractional topological states, in organic systems.

We measured angle-resolved photoelectron spectroscopy (ARPES) on large-scale uniform Ag-(BPhen)_3_ Kagome lattice on the surface of Ag(111). Unfortunately, we haven’t observed the prominent FBs (Supplementary Note 13), which may due to the very low carrier density in the Ag–(BPhen)_3_ Kagome lattice, supported by the calculated strong charge transfer from the Kagome MOF to the metal substrate (Fig. S20).

## METHODS

### Sample preparation

The Ag(111) single crystal was purchased from MaTeck, Germany, with an alignment with respect to the surface normal below 0.1°. A clean and structurally well-defined Ag(111) surface was prepared by bombarding with Ar^+^ ions and post annealing to 700 K. The precursor BBPPT was purchased from a commercial company and vapor-deposited from a commercial Kentax evaporator with quartz crucibles held at 453 K.

### STM/STS measurements

The experiments were performed in two UHV systems with background pressures better than 2 × 10^−10^ mbar. STM and STS measurements were obtained using SPM-INFINITY (Omicron GmbH), except for the K-doping measurements, which were performed on a Unisoku1500 instrument (Fig. 6, Figs S5, S6 and S8). The STS and d*I*/d*V* mapping were obtained by the standard lock-in method by applying an additional small AC voltage with a frequency of 713.3 Hz. The d*I*/d*V* spectra were collected by disrupting the feedback loop and sweeping the DC bias voltage. WSxM software was used for post processing of all STM data [47].

### K doping

Pure potassium (K, SAES Getters) was dosed through conventional resistance heating of a wire-type K dispenser after complete degassing. The K was calibrated and defined by applying a constant current (∼5.9 A) for a time interval of 0.5 min once. The pressure applied during the deposition of K atoms was maintained at a level lower than 2 × 10^−9^ mbar to ensure the purity of the alkali metal atoms.

### SRPES

SRPES experiments were conducted at the Catalysis and Surface Science End station of the National Synchrotron Radiation Laboratory, Hefei, China, which has been described previously [48]. The SRPE spectra were acquired at an emission angle of 50° with respect to the surface normal. Ag 3d, K 2p and N 1s spectra were collected with photon energies of 480, 380 and 480 eV, respectively. The chosen emission angle and photon energy were aimed at rendering the SRPES surface-sensitive.

### ARPES measurements

ARPES experiments were conducted at the Angle-Resolved Photoemission Spectroscopy (ARPES) Beamline of the National Synchrotron Radiation Laboratory, Hefei, China with a Scienta Omicron DA30-L analyzer and an *hν* = 30 eV light source. The samples were grown *in situ* and measured at *T* = 77 K (unless otherwise specified) with a background vacuum lower than 5 × 10^−11^ mbar.

### DFT calculations

DFT-based first-principles calculations of the freestanding Ag–(BPhen)_3_ Kagome lattice were performed with local-density approximation (LDA) [49] using the Vienna *ab initio* Simulation Package [50]. The energy cutoff of 520 eV was used for the plane-wave expansion. The 2 × 2 × 1 Monkhorst–Pack k-point grid [51] was used for Brillouin zone sampling. The unit cell of the Ag–(BPhen)_3_ Kagome lattice contains 243 atoms. For the structural optimization calculation, we fixed the lattice constants to experimentally confirmed values (*a* = *b* = 40.5 Å) and only relaxed the atomic positions. The energy and force convergence criteria were set to 10^−5^ eV and 0.01 eV/Å, respectively.

## Supplementary Material

nwaf574_Supplemental_File

## Data Availability

The data that support the findings of this study are available from the corresponding authors upon request.
