# Supplementary material for: Mott state of flat bands in a 2D metal–organic Kagome framework
Source: Natl Sci Rev. 2025 Dec 15;13(7):nwaf574. doi: 10.1093/nsr/nwaf574 (PMC13069685; doi:10.1093/nsr/nwaf574)
Supplement: nwaf574_Supplemental_File [file nwaf574_supplemental_file.pdf]

Supplementary Materials for

Mott state of flat bands in a two-dimensional metal−organic Kagome framework

Tianchen Qin[1,#], Xingyue Wang[2,#], Jia Wang[2,#], Xiaoyin Li[3,#], Sifan You[4,#], Zihan Wang[2], Junfa Zhu[1,*], Feng Liu[3,*], Lifeng Chi[4,*] and Minghu Pan[2,*]

[1]*National Synchrotron Radiation Laboratory, Department of Chemical Physics and Key Laboratory of Surface and Interface Chemistry and Energy Catalysis of Anhui Higher Education Institutes, University of Science and Technology of China, Hefei, Anhui 230029, P. R. China*

[2]*School of Physics and Information Technology, Shaanxi Normal University, Xi'an 710119, China.*

[3]*Department of Materials Science and Engineering, University of Utah, Salt Lake City, UT 84112, USA*

[4] *State Key Laboratory of Bioinspired Interfacial Materials Science, Institute of Functional Nano & Soft Materials (FUNSOM), Soochow University, 215123 Suzhou, China*

[#]These authors contributed equally to this work.

*E-mail: jfzhu@ustc.edu.cn; chilf@suda.edu.cn; minghupan@snnu.edu.cn; fliu@eng.utah.edu.

Contents

Supplementary Notes

Note S1. STM images evidence the large-scale uniformity of the *Ag-(BPhen)$_3$* Kagome lattice………………………………..……………………………….3
Note S2. Spectroscopic evidence for FBs existence on the *Ag-(BPhen)$_3$* Kagome lattice..5
Note S3. Spectroscopic evidence for the existence of Mott gap on the *Ag-(BPhen)$_3$* Kagome lattice…………………………………..…….……………6
Note S4. Defects in non-doped *Ag-(BPhen)$_3$* Kagome lattice.........................…....7
Note S5. Defects in K-doped *Ag-(BPhen)$_3$* Kagome lattice…………….…....….8
Note S6. A Defect and its dI/dV spectrum in K-doped *Ag-(BPhen)$_3$* Kagome lattice……………………………………………….……………………..9
Note S7. dI/dV maps of *Ag-(BPhen)$_3$* Kagome lattice at various biases……....10
Note S8. Trivial edge states of the *Ag-(BPhen)$_3$* Kagome lattice……………...…11
Note S9. Deduce the real gap depth from differential of measured I/V curves..14
Note S10. Simulation of temperature dependence of gaps with thermal broadening……………………………………………………………………….15
Note S11. An unusual gap state formed at the corner of the doped *Ag-(BPhen)$_3$* Kagome lattice after K doping…………………………………………………..17
Note S12. SRXPES measurements of K-doped *Ag-(BPhen)$_3$* Kagome lattice.19
Note S13. ARPES measurement on Ag-(BPhen)$_3$ Kagome lattice on Ag(111) surface……………………………………………………...………………22
Note S14. Optimized MOF structures on Ag(111) substrate and the calculated band structures..………………………………………………………………24
Note S15. Adsorption configuration of the Ag-(BPhen)$_3$ Kagome lattice on Ag(111)………………………………………………………………………….25
Note S16. A multistep sample covered by the single orientated Ag-(BPhen)$_3$ Kagome lattice………………………………….……………………..26
Note S17. High-resolution STM images revealing the structure of the Ag-(BPhen)$_3$ Kagome lattice………………………………………………………..27
Note S18. Calculated charge transfer…………………………………………….28
Note S19. The site-dependent Mott gap measurement of the Kagome lattice..29
Note S20. The STS, band structure and dI/dV mapping of the FB2………….30

Supplementary Figures and Tables

Fig. S1. Large-scale STM image showing the uniformity of the *Ag-(BPhen)$_3$* Kagome lattice……………………………………………………………………….2
Fig. S2. Spectroscopic signatures of a series of FBs measured across the *Ag-(BPhen)$_3$* Kagome lattice along different direction………………………………..4
Fig. S3. Spectroscopic signature of the Mott gap formed by the FB at E_F, measured across the *Ag-(BPhen)$_3$* Kagome lattice along different direction…6
Fig. S4. Defects in the *Ag-(BPhen)$_3$* Kagome lattice without K doping………..…7
Fig. S5. Defects in the *Ag-(BPhen)$_3$* Kagome lattice after <0.04 ML K doping…8
Fig. S6. Defects and dI/dV spectrum of the *Ag-(BPhen)$_3$* Kagome lattice after <0.04 ML K doping……………………..………………………….………………9
Fig. S7. dI/dV maps of the *Ag-(BPhen)$_3$* Kagome lattice at various biases…...10
Fig. S8. The edge state at the edge of the *Ag-(BPhen)$_3$* Kagome lattice after K doping measured at 9 K…………………………………………………………12
Fig. S9. Deduce the real gap depth from differential of measured I/V curves at two temperatures……..……………………………………………………………14
Fig. S10. Simulated dI/dV spectrum at various temperatures by considering thermal broadening effect……………………..…………………………………16
Fig. S11. An unusual gap state at the corner of the doped *Ag-(BPhen)$_3$* Kagome lattice after K doping measured at 1 K…………………………………………...18
Fig. S12. SRXPES measurements for *Ag 3d* and *K 2p* of K-doped *Ag-(BPhen)$_3$* Kagome lattice………………..………………………………………………19
Fig. S13. SRXPES measurements for *N 1s* of K-doped *Ag-(BPhen)$_3$* Kagome lattice……………………………………………………………………………..21
Fig. S14. ARPES measurement on Ag-(BPhen)$_3$ Kagome lattice on Ag(111) surface……………………………………………………………………………22
Fig. S15. Three configurations for MOF on Ag(111) and the corresponding bands……………………………………………………………………………..24
Fig. S16. The buckled-1 configuration of MOF and the bands………… …….24
Fig. S17. Adsorption configuration of the Ag-(BPhen)$_3$ Kagome lattice on Ag(111)……………………………………………………………………………...25
Fig. S18. STM image of a multistep sample covered by the Ag-(BPhen)$_3$ Kagome lattice……………………...……………………………………………26
Fig. S19. High-resolution STM images revealing the structure of the Ag-(BPhen)$_3$ Kagome lattice………………………………..…………………...27
Fig. S20. Calculated Charge density difference……………………………….28
Fig. S21. STS linecuts measured on the framework…….……………………..29

Fig. S22. The STS, band structure and dI/dV mapping of the FB2…………….30
Supplementary References……………………………………………………..31

Supplementary Results and Discussions

Supplementary Note 1. The large-scale uniformity of the *Ag-(BPhen)$_3$* Kagome lattice in STM images.

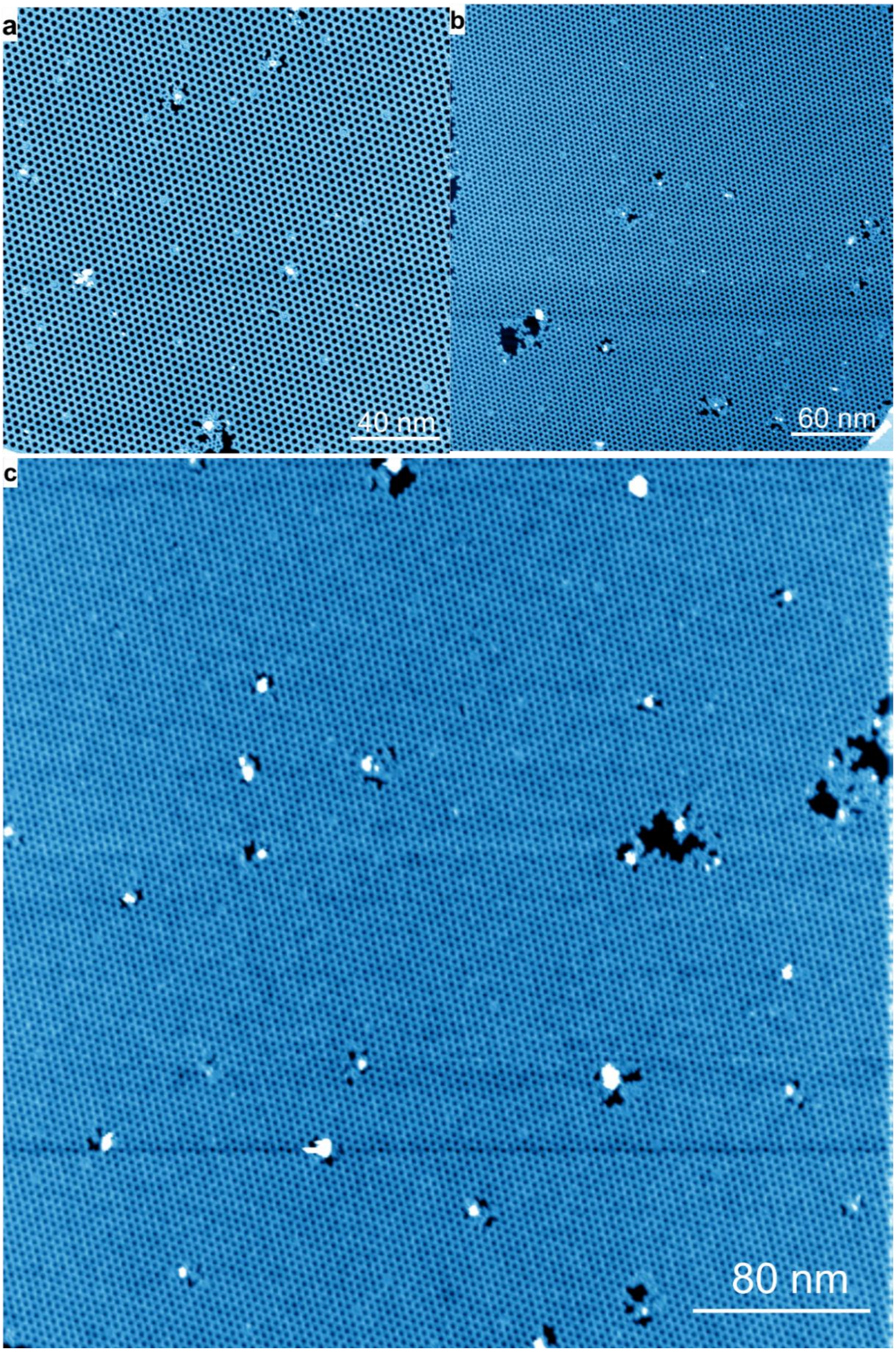

Supplementary Fig. 1 | Large-scale STM image showing the uniformity of the *Ag-(BPhen)₃* Kagome lattice. The image sizes are 200×200 (**a**), 300×300 (**b**), and 400×400 nm^2 (**c**), respectively, with V_B= -1000 mV and I_T= 100 pA.

Supplementary Note 2. Spectroscopic evidence for the existence of FBs in the *Ag-(BPhen)₃* Kagome lattice.

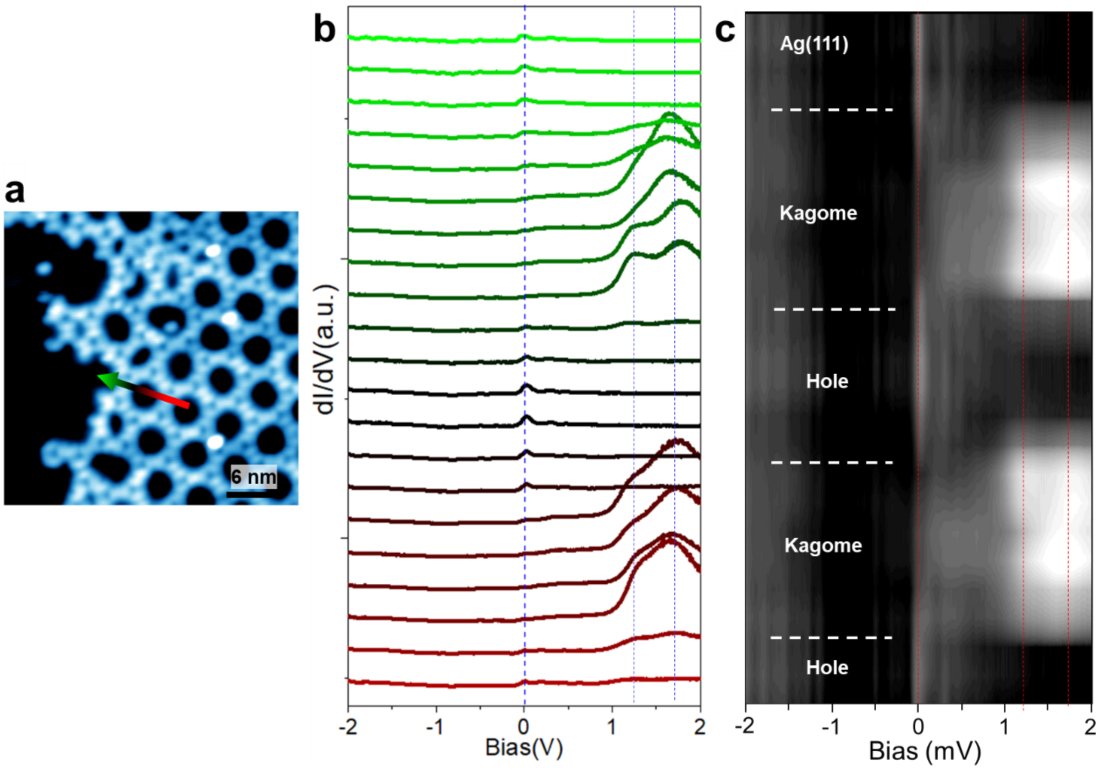

Supplementary Fig. 2 | Spectroscopic signatures of a series of FBs measured across the *Ag-(BPhen)$_3$* Kagome lattice along different directions. a, An STM image taken near the edge of the *Ag-(BPhen)$_3$* Kagome lattice. The measurements are V_B= -2200 mV and I_T= 100 pA. **b**, A line spectroscopic survey taken along the arrowed line in panel **a**. All dI/dV spectra were measured with V_B= -2000 mV, I_T= 500 pA and a bias modulation of 25 mV at 9.2 K. **c**, A grayscale plot of site-dependent dI/dV spectra. From the top of the panel, the LDOS varies from the Ag(111) surface to the Kagome lattice, in which the DOS peaks of FBs only appear on the *Ag-(BPhen)$_3$* Kagome lattice with DOS peaks of FBs located at high positive energies.

Supplementary Note 3. Spectroscopic evidence for the existence of Mott gap in the *Ag-(BPhen)₃* Kagome lattice

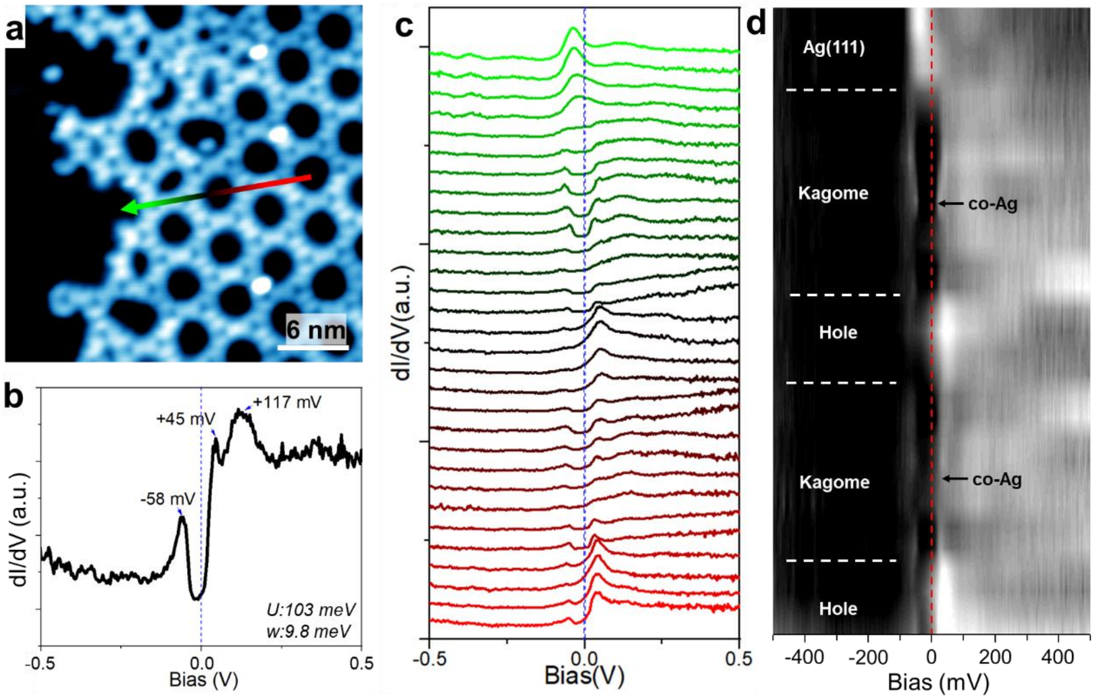

Supplementary Fig. 3 | Spectroscopic signature of the Mott gap formed by the FB at E_F, measured across the *Ag-(BPhen)$_3$* Kagome lattice along different direction. a, An STM image taken near the edge of the *Ag-(BPhen)$_3$* Kagome lattice. The measurements are V_B= -2200 mV and I_T= 100 pA. **b**, A point STS measured on the Kagome lattice showing the formation of a UHB/LHB and a Mott gap of approximately ~103 meV at E_F. **c**, A line spectroscopic survey taken along the arrowed line in panel **a**. All dI/dV spectra were measured with V_B= -500 mV, I_T= 1000 pA and a bias modulation of 5 mV. **d**, A gray scale plot of site-dependent dI/dV spectra. From the top of the panel, the LDOS varies from the Ag(111) surface to the Kagome lattice, in which the UHB/LHB bands only appear in the *Ag-(BPhen)$_3$* Kagome lattice with a well-defined gap. Mott gap on benzene bone is slightly larger than that measured on the site of coordinated Ag, which can be seen in here. It might indicate a leaking effect at the site of coordinated Ag.

Supplementary Note 4. *(BPhen)₃*-embedded defects in non-doped *Ag-(BPhen)₃* Kagome lattice.

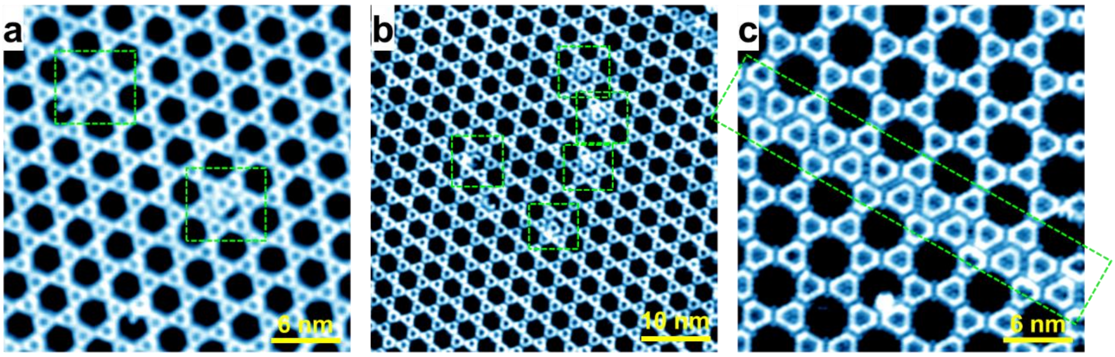

Supplementary Fig. 4 l BBPPT-embedded defects in the original *Ag-(BPhen)₃* Kagome lattice without potassium doping. **a**, A pair of *(BPhen)₃*-embedded defects in a perfect Kagome lattice. The image size is 30×30 nm^2 with a V_B of -1000 mV and an I_T of 80 pA. **b**, Four individual *(BPhen)₃*-embedded defects. The image size is 50×50 nm^2 with V_B= -700 mV and I_T= 100 pA. **c**, A chain of *(BPhen)₃*-embedded defects form a domain boundary. The image size is 30×30 nm^2 with a V_B of -900 mV and an I_T of 100 pA.

Supplementary Note 5. Defects in K-doped *Ag-(BPhen)$_3$* Kagome lattice.

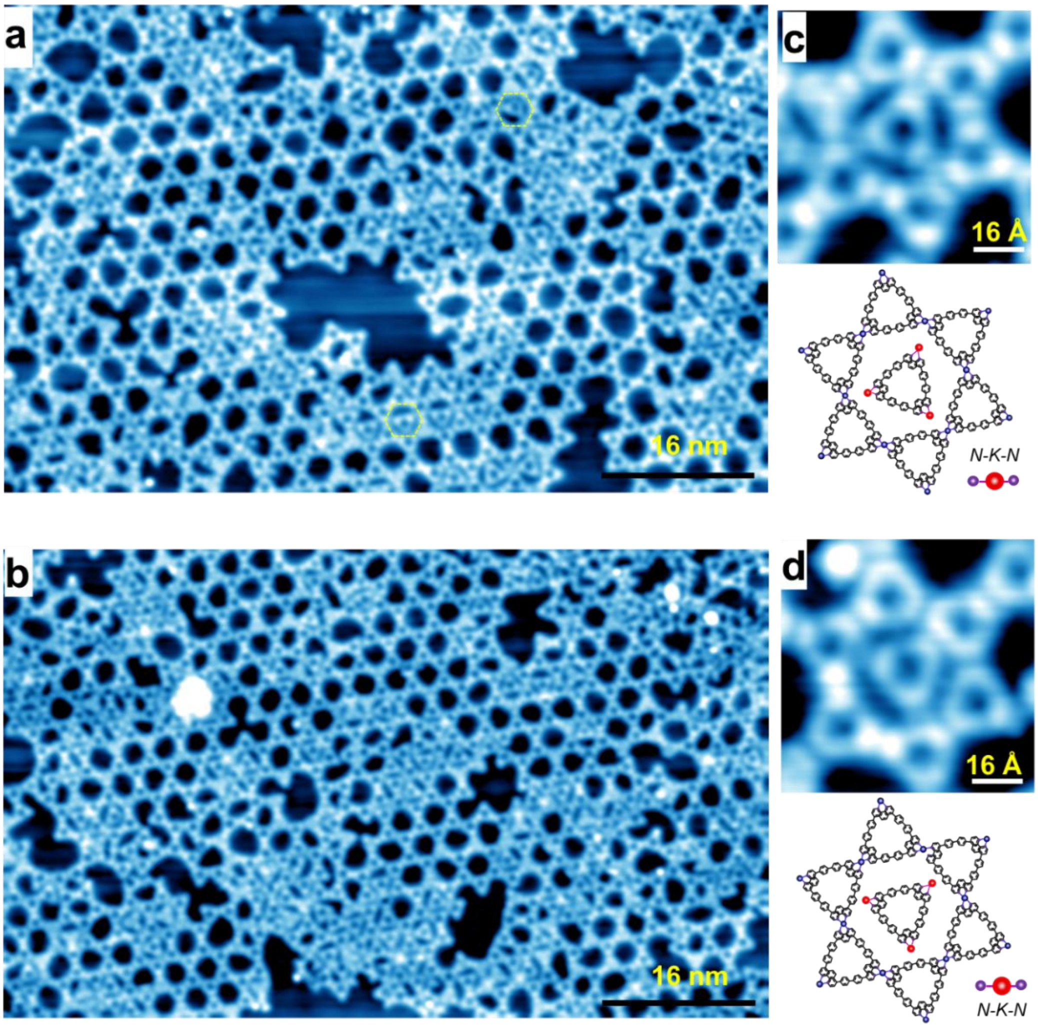

Supplementary Data Fig. 5 | (BPhen)$_3$-embedded defects in the *Ag-(BPhen)$_3$* Kagome lattice after <0.04 ML potassium doping. a-b, Topographic images showing the appearance of numerous (BPhen)$_3$-embedded defects after K doping. (**a**) The image size is 80×51 nm^2 with V_B= -200 mV and I_T= 10 pA. (**b**) The image size is 100×61 nm^2 with V_B= -200 mV and I_T= 10 pA. **c-d**, High-resolution images of two different (BPhen)$_3$-embedded defects with different orientations of the inside-embedded (BPhen)$_3$ molecule (lower) and the proposed structural models for two different (BPhen)$_3$-embedded defects. The image size is 8×8 nm^2 with a V_B=-200 mV and an I_T= 10 pA. Here, the embedded (BPhen)$_3$ is actually terminated by 2-fold N-K bonds, instead of 4-fold N-Ag bonds, as demonstrated by our recent work [1].

Supplementary Note 6. A (BPhen)$_3$-embedded defect and its dI/dV spectrum in K-doped *Ag-(BPhen)$_3$* Kagome lattice.

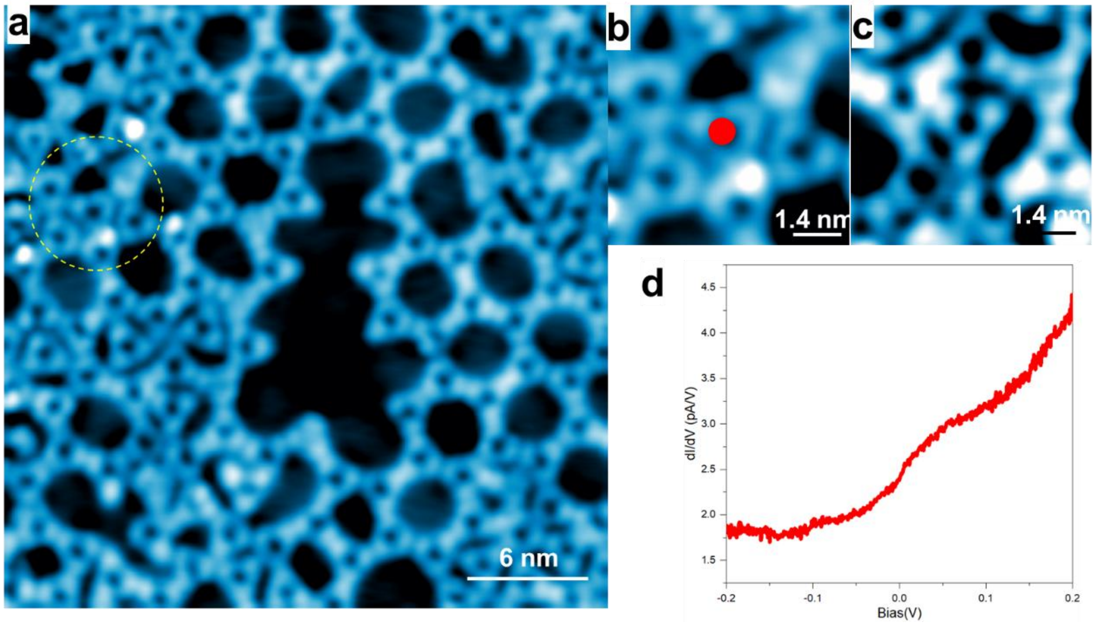

Supplementary Fig. 6 | (BPhen)$_3$-embedded defects and dI/dV spectrum of the *Ag-(BPhen)$_3$* Kagome lattice after <0.04 ML potassium doping. a, Topographic images showing the appearance of numerous (BPhen)$_3$-embedded defects after K doping. The image size is 30×30 nm^2 with a V_B of +60 mV and an I_T of 20 pA measured at 4 K. **b-c**, High-resolution images of two different (BPhen)$_3$-embedded defects with different locations of the inside-embedded (BPhen)$_3$ molecule. The image size is 7×7 nm^2 with V_B=+60 mV and I_T= 20 pA. **d**, dI/dV spectrum measured at the center of the defect, shown as a red spot in panel **b**, which indicates a metallic behavior suggesting doping-induced Mott metal-insulator transitions (MITs).

Supplementary Note 7. dI/dV maps of the undoped *Ag-(BPhen)₃* Kagome lattice at various biases.

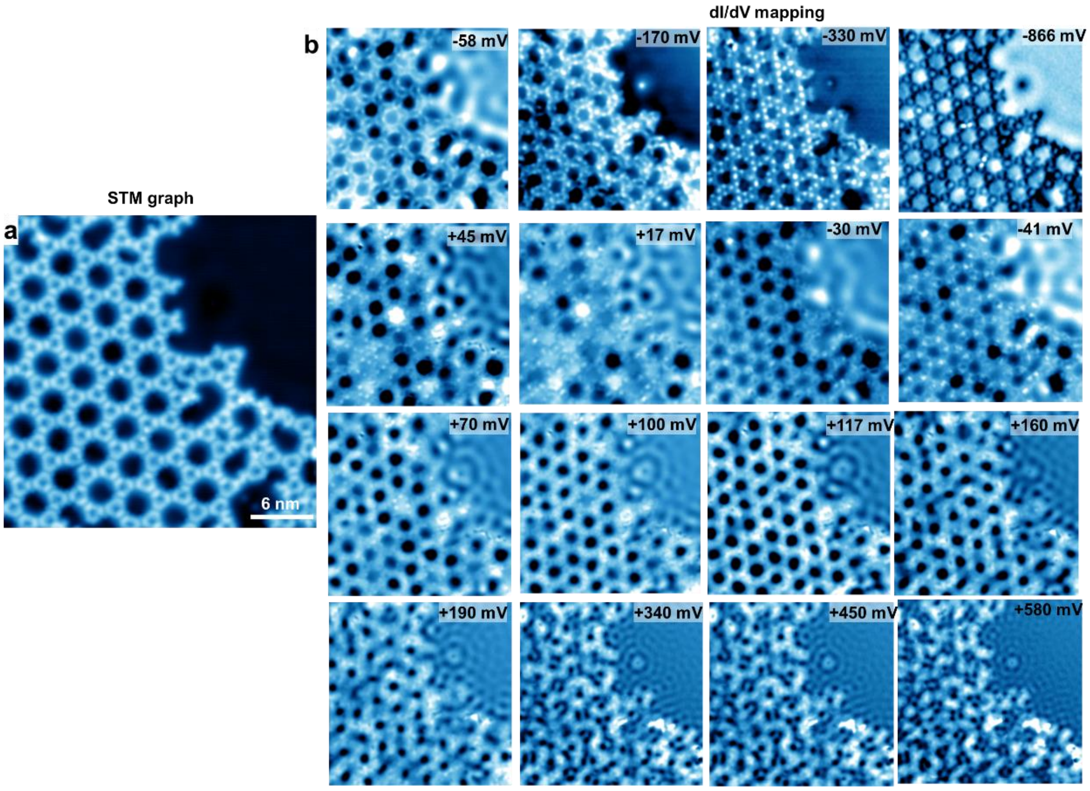

Supplementary Fig. 7 | dI/dV maps of the original *Ag-(BPhen)*$_3$ Kagome lattice at various biases. a, Topographic image**; b,** dI/dV maps taken at various biases**.** All dI/dV maps were measured with an I_T of 500 pA and a bias modulation of 25 mV.

Supplementary Note 8. The trivial edge states observed at the edge of the *Ag-(BPhen)$_3$* Kagome lattice

Dirac and flat bands have been predicted in various two-dimensional (2D) MOFs [2], forming topological insulator (TI) and Chern insulator phases [3-5]. However, the tiny spin-orbit coupling (SOC) in organic materials makes it extremely difficult to detect topological boundary states within the SOC gap (the SOC-induced gap opening of the degenerate point (Γ point) between the flat band and the dispersive band.) [6-8]. Obviously, the SOC gap of this Kagome lattice is very small, which is estimated in the range of few meV. Previous work revealed that topologically protected edge or corner states may emerge in opened gaps in electronic breathing Kagome lattices [9-12].

We therefore performed tunneling spectroscopic measurements across the edge of the *Ag-(BPhen)$_3$* Kagome lattice. **Figure S8b** shows a series of spectra acquired along the colored arrowed line in **Fig. S8a** across the edge of the MOF island. The green and red curves (**Fig. S8c**) represent the dI/dV spectra taken at the edge and internal region (far away from the edge), respectively. Importantly, enhanced DOS are clearly observed in the energy range from -500 to 0 mV in the spectra (represented as shadow areas) taken at the edge. In order to remove the electronic DOS originating from the bulk band, we extracted representative spectra measured at the internal from the edge of the *Ag-(BPhen)$_3$* Kagome lattice. As illustrated in the inset panel of **Fig. S8d**, the curve represents the electronic DOS only contributed by the edge states, showing a larger edge DOS in the negative biases (especially at -330 mV, -170 mV and -30 mV), marked with a cyan shadow area. The spectroscopy of edge states, shown in **Fig. S8c**, exhibits the energies of edge states at approximately -330, -170 and -30 meV, located in between -0.6 eV and 0 eV, which are indicative of non-topological edge features.

By taking dI/dV mapping at the energies of the edge states, we can distinguish

the spatial distributions of the edge states of the Kagome lattices. **Figures S8e-f** show the dI/dV maps taken at an energy of -330 meV in the energy range of the edge states. In addition, the dI/dV maps taken at an energy of -170 meV also show prominent edge states (**Suppl. Fig. S7**). However, the dI/dV map at -30 mV does not show any extra brightness of edge states, and the reason is still unclear.

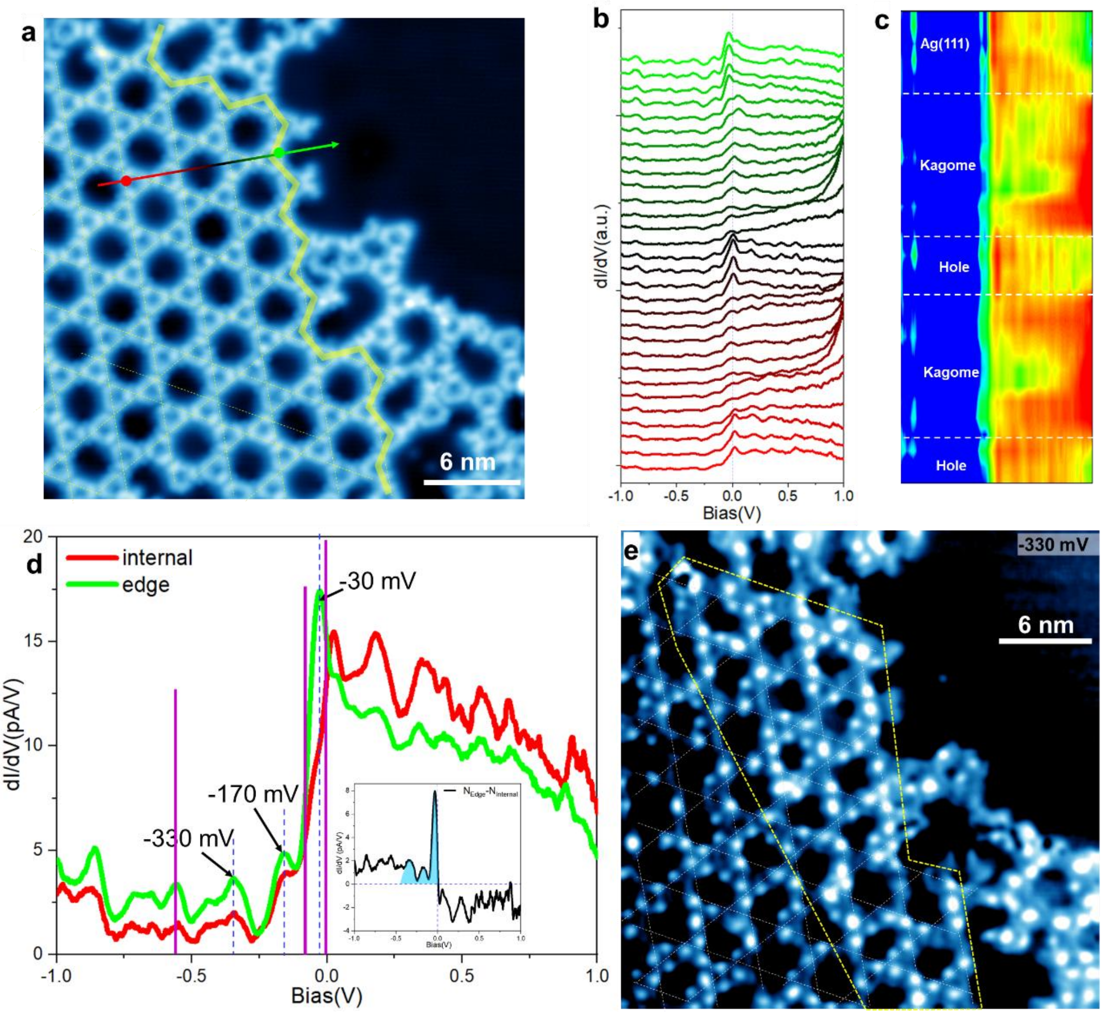

Supplementary Fig. 8 | The edge state at the edge of the *Ag-(BPhen)₃* Kagome lattice after K doping measured at 9 K. a, An STM image taken at the boundary of the *Ag-(BPhen)*$_3$ Kagome lattice. The image size is 30×30 nm^2 with V_B= +1000 V and I_T= 100 pA. **b**, A series of dI/dV spectra measured along the colored arrow line in panel **a** near the edge of the *Ag-(BPhen)*$_3$ Kagome lattice. All dI/dV spectra were measured with V_B= -1000 V, I_T= 1000 pA and a bias modulation of 15 mV. **c**, A color-scale plot of site-dependent dI/dV spectra. The measurements are V_B=-1000 mV, I_T=1000 nA and a bias modulation of 15 mV. From the top of the panel, the LDOS varies from the Ag(111) surface to the Kagome lattice. **d**, Point spectra taken near the edge (green) and inside (red),

as shown in panel **a**, respectively. Purple vertical bars show three FBs (purple short bars), located at 0, -0.1 and -0.6 eV; (Inset, low) The edge state (N_{Edge}-$N_{Internal}$), deduced from subtracting the internal curve from the edge curve to remove the effect of Ag surface states, shows a larger edge DOS in the negative biases (especially at -330 mV, -170 mV and -30 mV), marked with a cyan shadow area. Such edge states are formed in between -0.6 eV and 0.1 eV, showing a dual-V shape. **e**, dI/dV maps at the energy of the edge states (-330 meV).

Supplementary Note 9. Deduce the real gap depth from the differential of measured I/V curves.

As we know, dI/dV spectrum measured by lock-in method usually includes an uncertain background due to RF and other noise. In order to obtain real gap depth, we simultaneously measured current/voltage (I/V) curves with dI/dV spectrum. We perform numerical differential for measured I/V curves to obtain gap depths for each temperature.

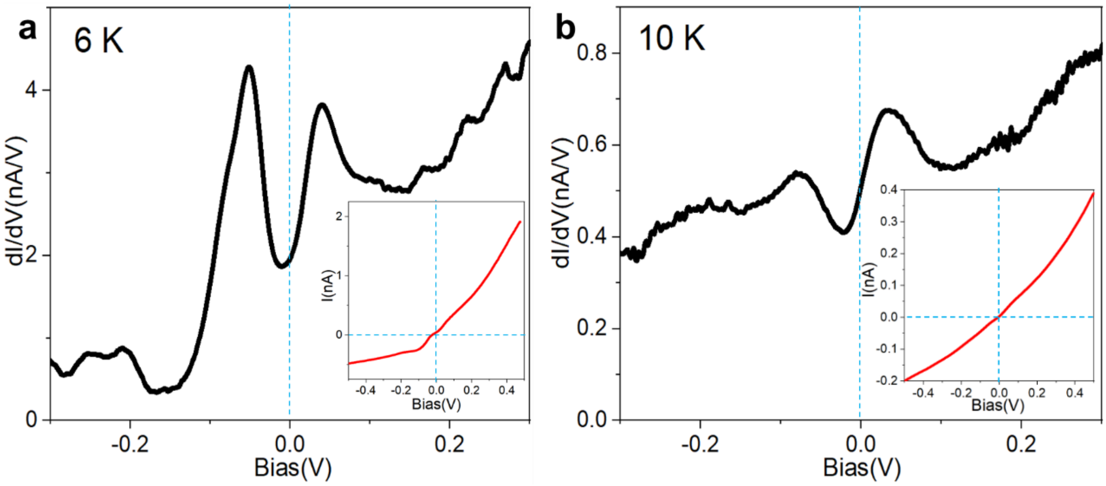

Supplementary Fig. 9 | Deduce the real gap depth from the differential of measured I/V curves at two temperatures, 6 K and 10 K. a-b, Numerical differential for measured I/V curves at 6 K and 10 K, respectively. (Inset) Simultaneously measured current/voltage (I/V) curves. All I/V curves were measured with V_B= -500 mV, I_T= 500 pA.

Supplementary Note 10. Simulation of temperature dependence of energy gap with thermal broadening

In order to confirm that the temperature dependence of the energy gap at zero bias is not just an effect of thermal broadening, we have performed a thermal broadening simulation:

$$\frac{dI}{dV}(eV) \propto \int_{-\infty}^{\infty} \rho(E)\left(\frac{d}{dV}F(E-eV,T)\right)dE$$

where $\rho(E)$ is the zero-temperature DOS and $F(E,T)$ is the Fermi distribution function. Since we only measured the 2 K tunneling spectrum and the thermal broadening at 2 K is small, we constructed a dI/dV spectrum as DOS at 0 K ($\rho_0(E)$), then apply the thermal broadening of 2 K in the simulation, and compare with the experimental data. By fine tuning the $\rho_0(E)$ to achieve a reasonably good fitting (**Fig. 4b**), we took the $\rho_0(E)$ as $\rho(E)$ and calculated a series of dI/dV spectrum at various temperatures (**Fig. S10a**). Next, the red curve in **Fig. 4c** was generated by using the F (E, T) at T = 10 K, and one sees that the measured and simulated spectrum at 10 K clearly do not match, which means that this is not just an effect of thermal broadening. Considering the real thermal broadening $4K_BT$ (composed of ~$2K_BT$ for the sample and ~$2K_BT$ for the tip) [13] instead of 1 K_BT, we also simulated dI/dV spectrum with $4k_BT$ smearing at various temperatures (**Fig. S10b**). Moreover, the simulated thermal broadening of the gap depth ΔD and the experimentally measured values as a function of temperature are shown in **Fig. 4d**. Here, the gap depth ΔD is defined by the ratio of gap depth (ΔD_G, cyan arrow) and peak height (ΔH_P, blue arrow) in **Fig. 4a**.

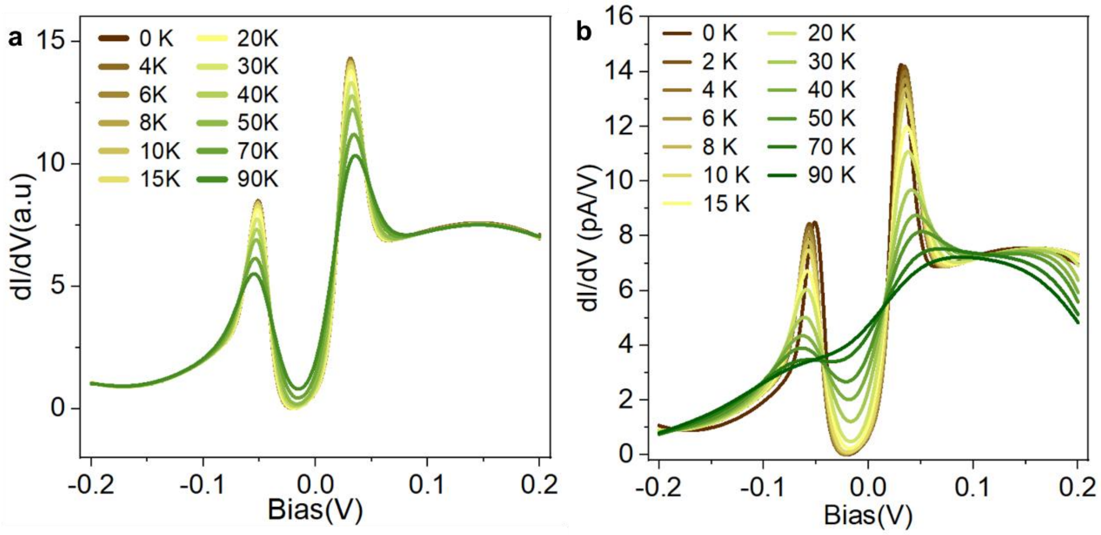

Supplementary Fig. 10 | Simulated dI/dV spectrum at various temperatures by considering thermal broadening effect. a, Simulation by considering thermal broadening of $1K_BT$. **b**, Simulation by considering thermal broadening of $4K_BT$.

Supplementary Note 11. An unusual gap state formed at the corner of the doped *Ag-(BPhen)*$_3$ Kagome lattice after K doping.

With electron doping from K, formation of corner state of the *Ag-(BPhen)*$_3$ Kagome lattice is observed. We measured a series of dI/dV spectra at the corner of the destructive *Ag-(BPhen)*$_3$ Kagome lattice after K doping at 1 K (**Fig. S11b**). A symmetric gap feature is developed in the dI/dV curves near the corner. The size of this gap is approximately 15 meV (**Fig. S11c**), which was not observed in the original *Ag-(BPhen)*$_3$ Kagome lattice (see **Figs. S2** and **S3**). By applying a perpendicular magnetic field up to 1 Tesla (**Fig. S11d**), the shape of this gap remains almost the same. The existence of topological corner states in 2D MOFs and the related intriguing higher-order nontrivial topology in the energy window between two Kagome bands or between Dirac bands have been recently demonstrated both experimentally and theoretically [[12,14]]. We speculate such gap feature could arise from strong correlation of electrons confined at the corner.

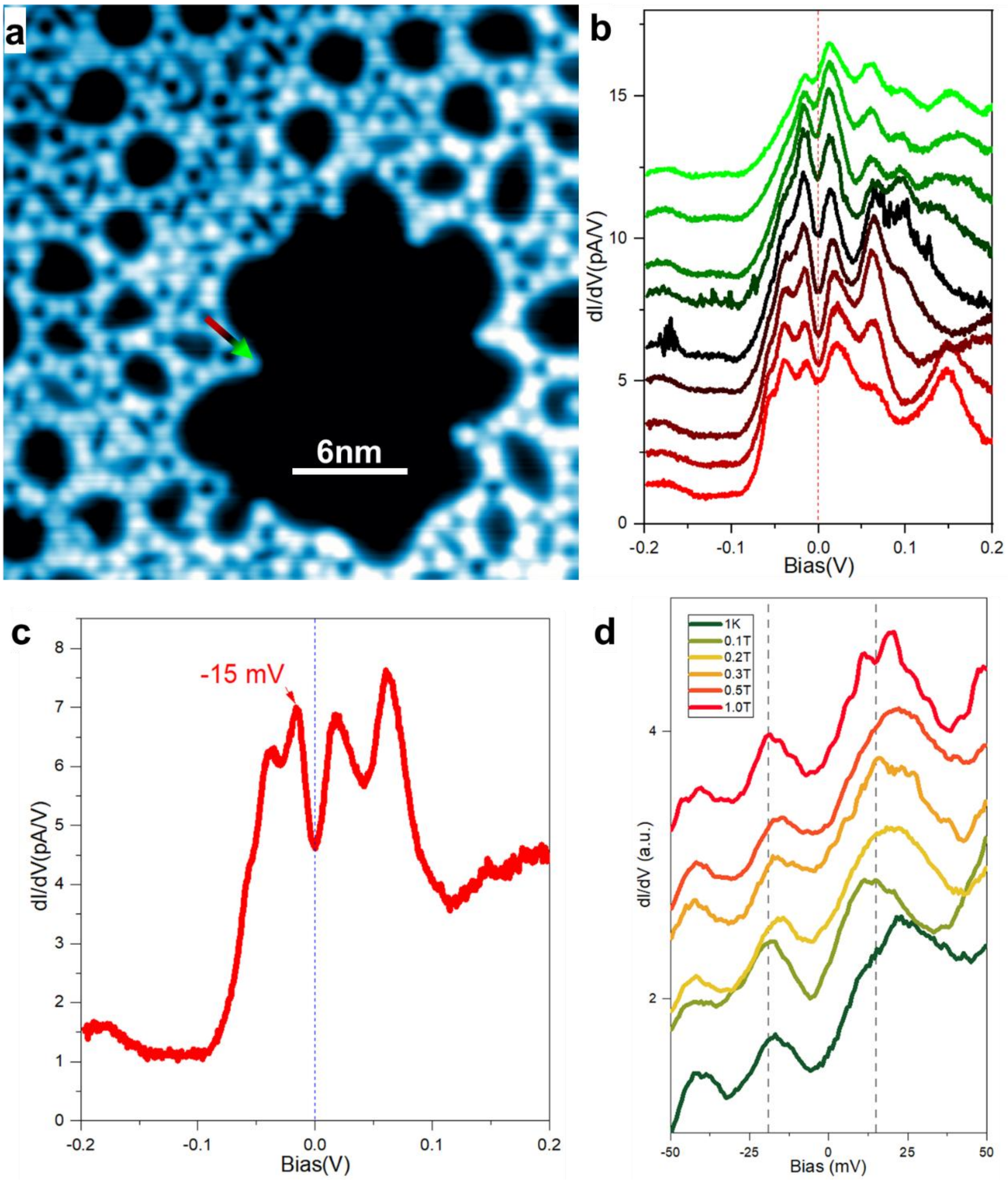

Supplementary Fig. 11 | an unusual gap state at the corner of the doped *Ag-(BPhen)*$_3$ Kagome lattice after K doping measured at 1 K. a, Topological STM image of a region with a destructive *Ag-(BPhen)*$_3$ Kagome lattice. The image size is 30×30 nm^2 with V_B= +300 mV and I_T= 10 pA. **b**, a series of dI/dV spectra measured along the colored arrow line in panel **a** at the corner of the destructive *Ag-(BPhen)*$_3$ Kagome lattice. Multi-DOS peaks appear around the E_F.**c.**, a typical dI/dV spectrum showing symmetric gap features at E_F, with a gap size of approximately 15 meV. d, Variation of the gap feature under a varying perpendicular magnetic field of 0, 0.1, 0.2, 0.3 and 0.5 Tesla. The dI/dV spectra were measured with V_B= +300 mV, I_T=200 pA and a bias modulation of 2 mV at a temperature of 1 K.

Supplementary Note 12. Synchrotron radiation X-ray photoemission spectroscopy (SRXPES) measurements of K-doped *Ag-(BPhen)$_3$* Kagome lattice

To investigate the impact of K dopant on the coordination and bonding patterns within a Kagome lattice, we conducted synchrotron radiation photoemission spectroscopy (SRPES) measurements. From the Ag 3d spectra (**Fig. S12a**), we observed that the signals corresponding to the Ag−N bond increase with the increasing K doping concentration. The K 2p spectra (**Fig. S12b**) showed that when the concentration of K dopant was more than 0.5 ML, the signal of K−N remained constant, indicating that the coordination bond has reached saturation. Additionally, the N 1s spectra of pure Kagome lattice exhibited two signals, attributed to the N−C and N−Ag bonds (**Fig. S13a**). However, with K doping beyond 0.5 ML, a new signal with higher binding energy appeared. Based on theoretically stoichiometric calculations, we assigned this new signal to N−K. We propose that two nitrogen atoms at each phenanthroline site form two new coordination bonds with one K atom within the Kagome monolayer, while another K atom above the Kagome monolayer forms two additional coordination bonds with two other N atoms. Therefore, we conclude that the transformation of the Kagome lattice is due to the stronger electron-donating ability of K, which forms more stable coordination bonds with N, thereby competing with Ag atoms and replacing the previously four-fold N−Ag coordination bonds. The significantly larger atomic radius of K compared to Ag disrupts the previously well-matched four-fold N−Ag coordinated Kagome lattice with the Ag(111) substrate. Furthermore, K atoms tend to form two-fold N−K coordination bonds with N instead of four-fold coordination bonds, preventing the formation of a new Kagome structure. All the SRPES results can also be found in ref. [[1]].

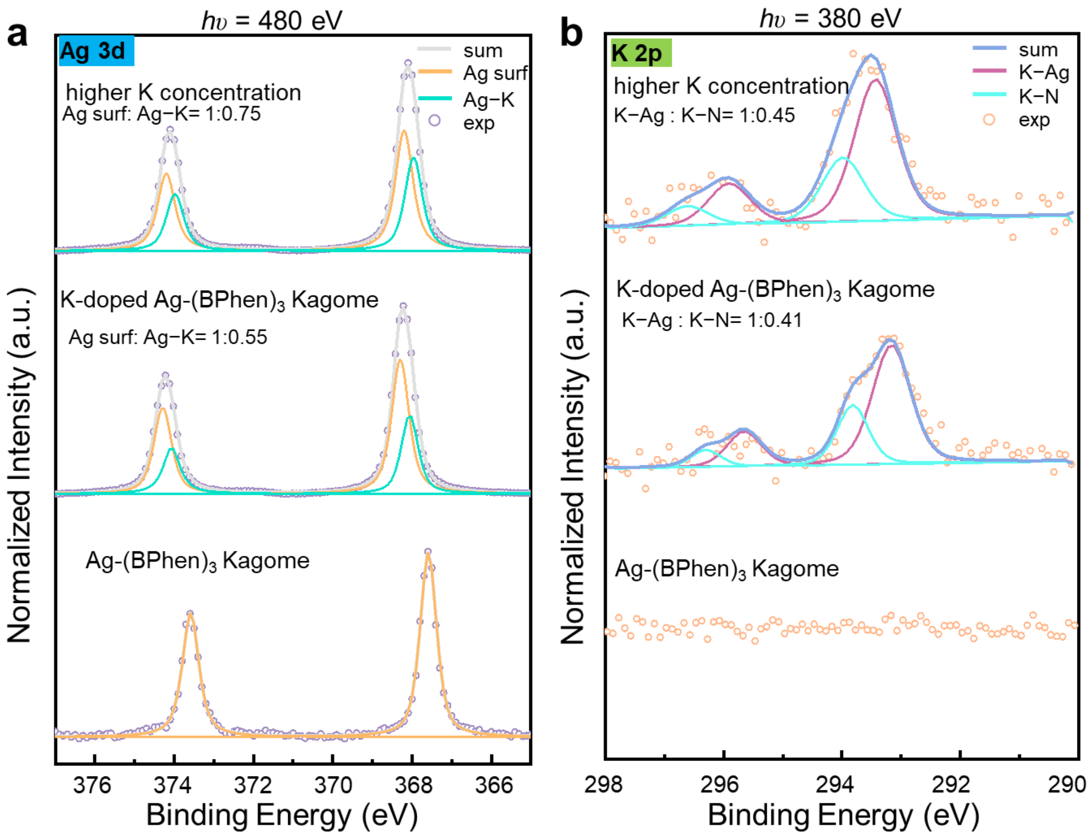

Supplementary Fig. 12 I SRXPES measurements for *Ag 3d* and *K 2p* of K-doped *Ag-(BPhen)₃* Kagome lattice. SRXPES spectra of (**a**) *Ag 3d* and (**b**) *K 2p* recorded at different at different K coverages measured with different photon energies.

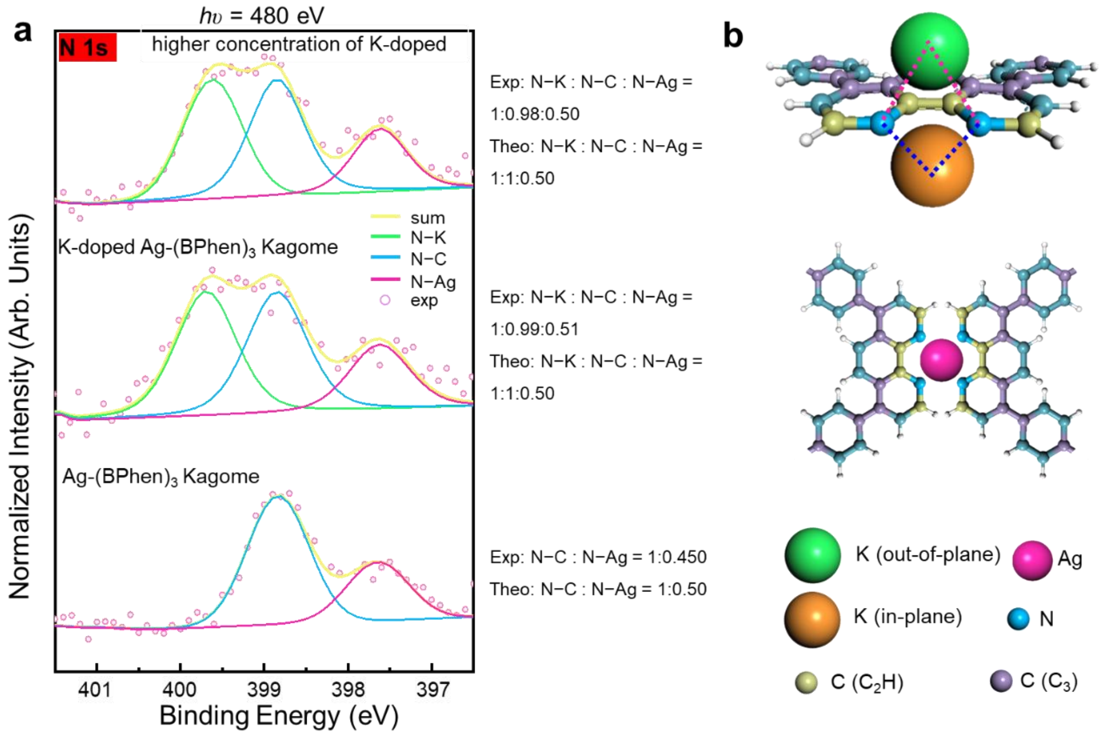

Supplementary Fig. 13 | SRXPES measurements for *N 1s* of K-doped *Ag-(BPhen)$_3$* Kagome lattice. a, SRXPES spectra of N 1s recorded at different K coverages. **b**, Molecular models of K configurations at different K coverages. Different atoms are depicted in different colors to illustrate their chemical environments.

Supplementary Note 13. Angle-resolved photoelectron spectroscopy (ARPES) measurement on Ag-(BPhen)$_3$ Kagome lattice on Ag(111) surface.

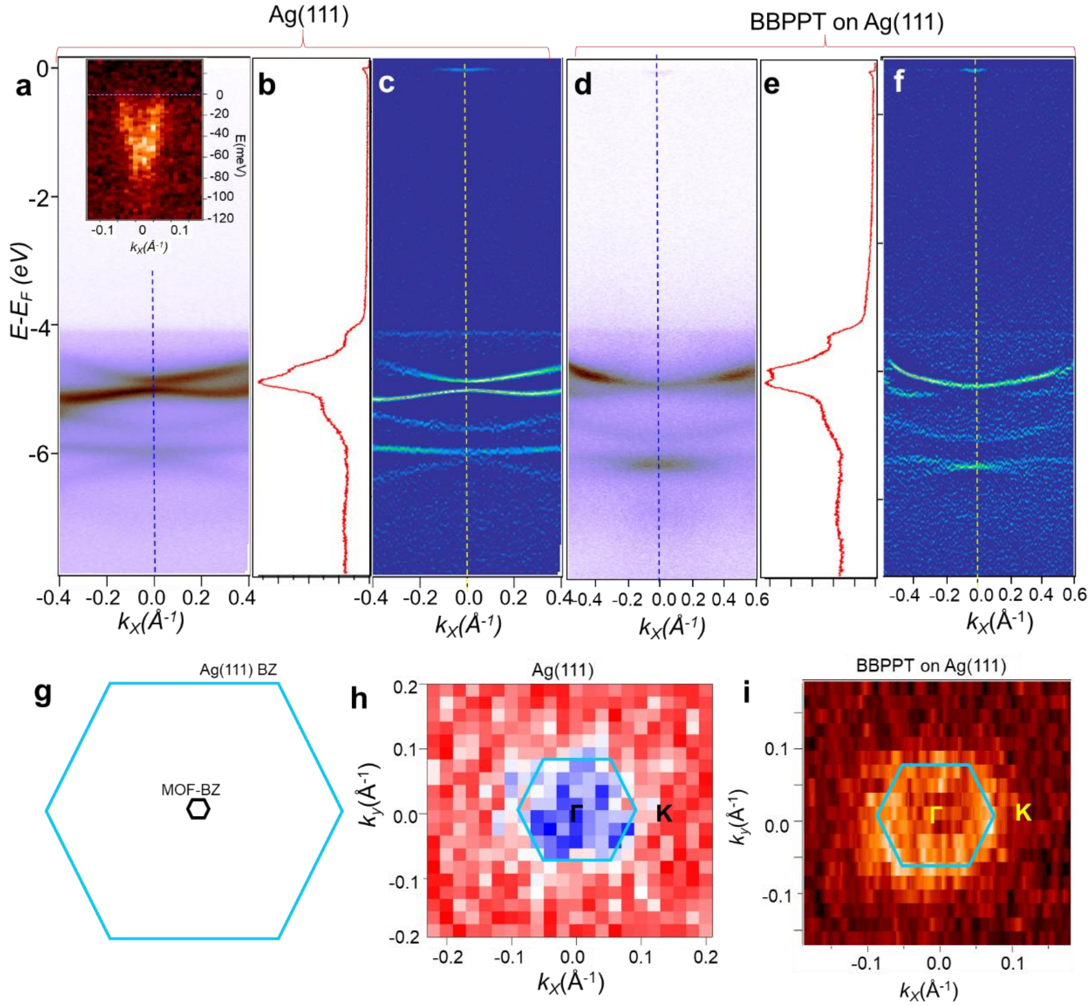

Supplementary Fig. 14 | ARPES measurement on Ag-(BPhen)$_3$ Kagome lattice on Ag(111) surface. a-c, High-resolution ARPES spectra **(a)**, integrated DOS from the ARPES (**b**) and second-derivative intensity plot (**c**) of Ag(111) surface. **d-f,** High-resolution ARPES spectra **(d)**, integrated DOS from the ARPES (**e**) and second-derivative intensity plot (**f**) of Ag-(BPhen)$_3$ Kagome lattice. **g,** The relationship between the BZs of Ag(111) surface and Ag-(BPhen)$_3$ MOF lattice. Note that the lattice constant of Ag-(BPhen)$_3$ MOF (40.5 Å) is about 13.78 times of that of Ag (2.94 Å) and the lattice vectors a_1 and a_2 are aligned parallel with the graphite lattice. **h-i**, The CEC maps measured at 0.0 eV and 77 K for Ag(111) surface and Ag-(BPhen)$_3$ MOF lattice, respectively. Blue hexagons indicate Fermi pockets of surface states in (**h**) and BZ of Ag-(BPhen)$_3$ MOF in (**i**). Considering the size of the Ag(111) surface Brillouin zone (BZ) (1.5 Å^{-1} from Γ to K) [15], the deduced BZ of Ag-(BPhen)$_3$ MOF is about 0.108 Å^{-1}.

Supplementary Note 14. Optimized MOF structures with substrate and the calculated band.

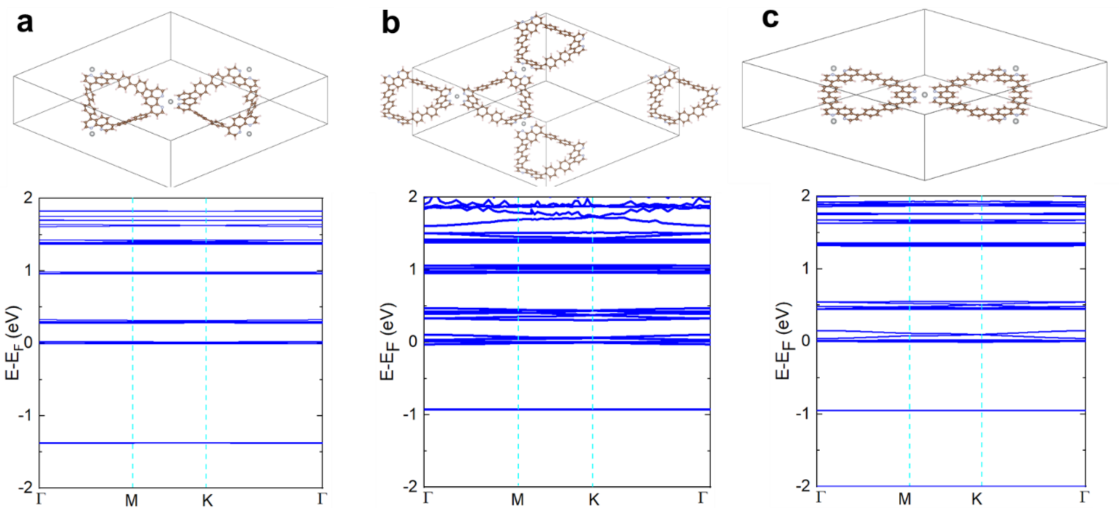

Supplementary Fig. 15 l a, Side view of the buckled-1 configuration (C6 symmetry) of MOF on Ag(111) and the corresponding band structures. The energy of this structure is -1715.782 eV/uc, the lowest. **b**, Side view of the buckled-2 configuration (C3v symmetry) of MOF on Ag(111) and the corresponding band structures. The energy of this structure is =-1714.676 eV/uc. **c**, Side view of the flat configuration (D6h symmetry) of MOF on Ag(111) and the corresponding band structures. The energy of this structure is =-1707.596 eV/uc.

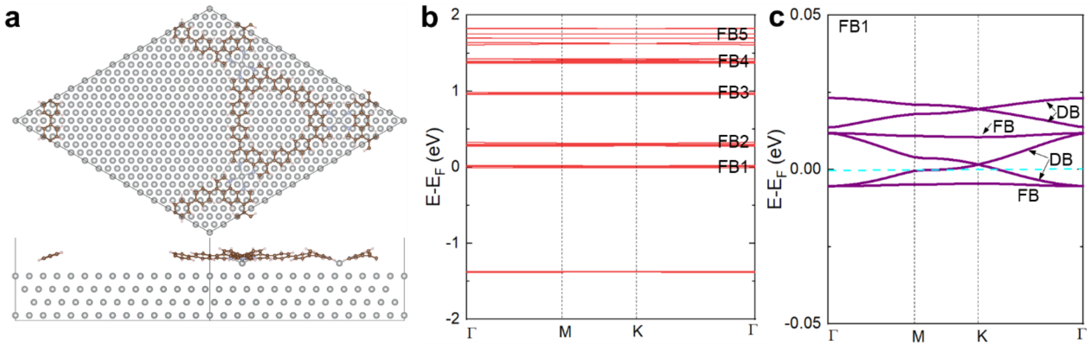

Supplementary Fig. 16 l a, Top and side view of the buckled-1 configuration of MOF on Ag(111). **b**, The corresponding band structures in the energy range from -2 to +2 eV. **c**, the bands near E_F.

Supplementary Note 15. Adsorption configuration of the Ag-(BPhen)$_3$ Kagome lattice on Ag(111).

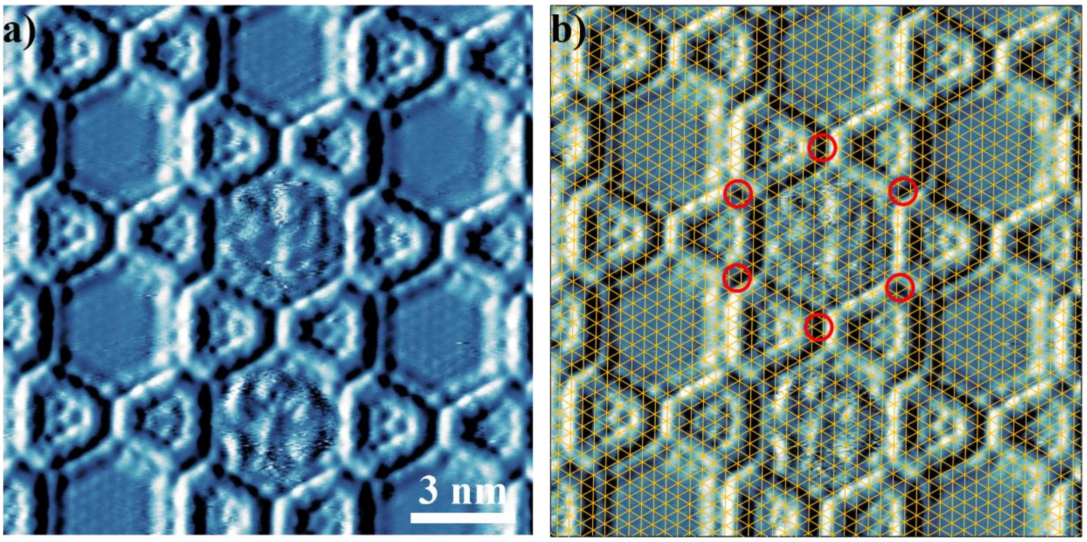

Supplementary Fig. 17 | Adsorption configuration of the Ag-(BPhen)$_3$ Kagome lattice on Ag(111). (a) STM image of the Ag-(BPhen)$_3$ Kagome lattice along with the visible atomic-phase structure of Ag(111) substrate. (b) Orange grid of the substrate lattice covered on (a). The crossings of the grid represent the positions of the Ag atoms in the substrate. The locations of the coordinated Ag adatoms in the Ag-(BPhen)$_3$ Kagome lattice are highlighted by red circles. Tunneling parameters: V_B =−920.1 mV, I_T = 101 pA.

Supplementary Note 16. A multistep sample covered by the single orientated Ag-(BPhen)$_3$ Kagome lattice.

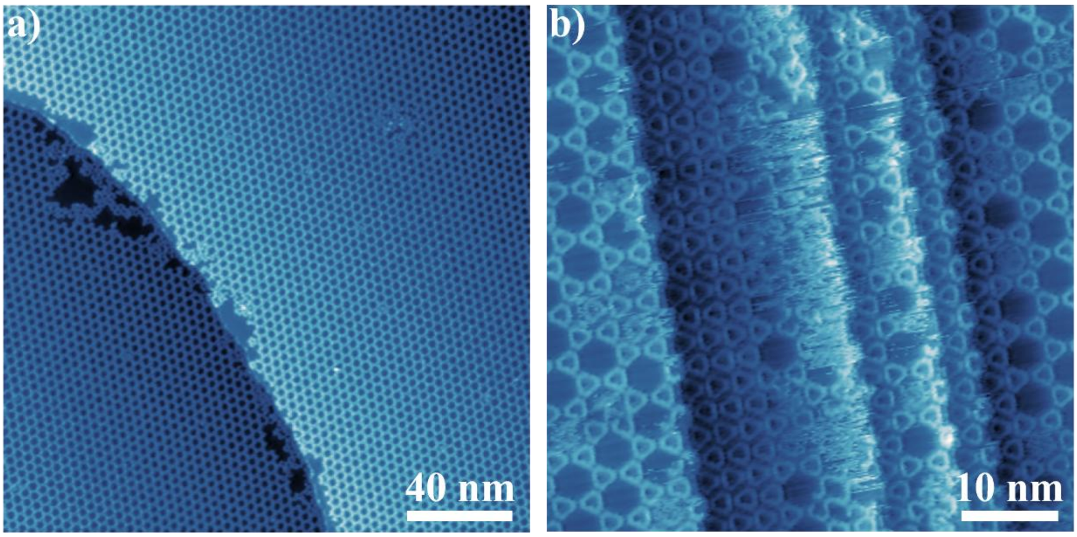

Supplementary Fig. 18 | STM image of a multistep sample covered by the Ag-(BPhen)$_3$ Kagome lattice. Tunneling parameters: (a) V_B =−2018 mV, I_T = 100 pA; (b) V_B =−1010 mV, I_T = 120 pA.

Supplementary Note 17. High-resolution STM images revealing the structure of the Ag-(BPhen)$_3$ Kagome lattice.

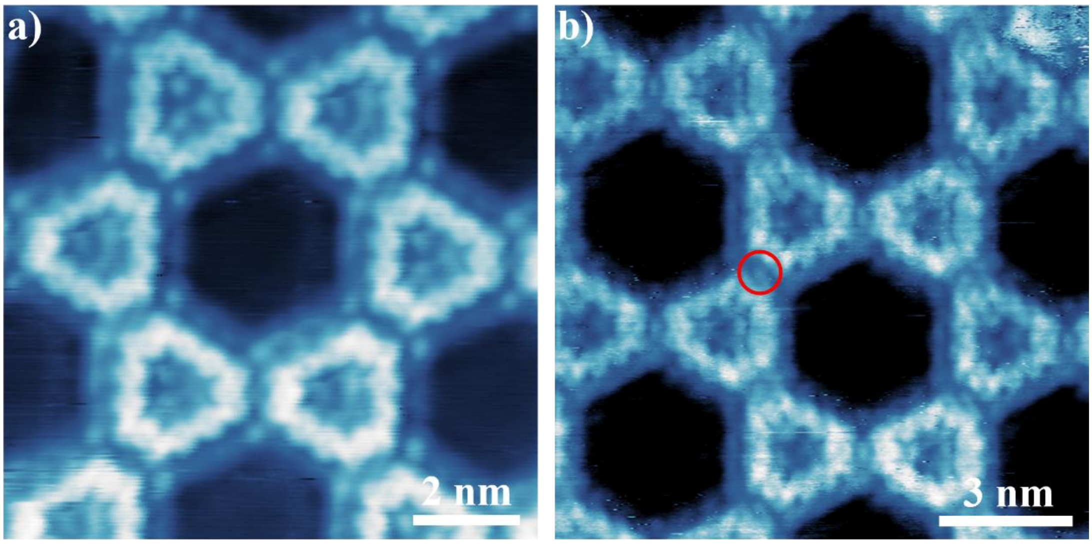

Supplementary Fig. 19 | High-resolution STM images revealing the structure of the Ag-(BPhen)$_3$ Kagome lattice. The locations of the coordinated Ag adatoms in the Ag-(BPhen)$_3$ Kagome lattice are highlighted by red circle. Tunneling parameters: (a) V_B =−1010 mV, I_T = 80 pA; (b) V_B =−1010 mV, I_T = 120 pA.

Supplementary Note 18. Calculated charge transfer

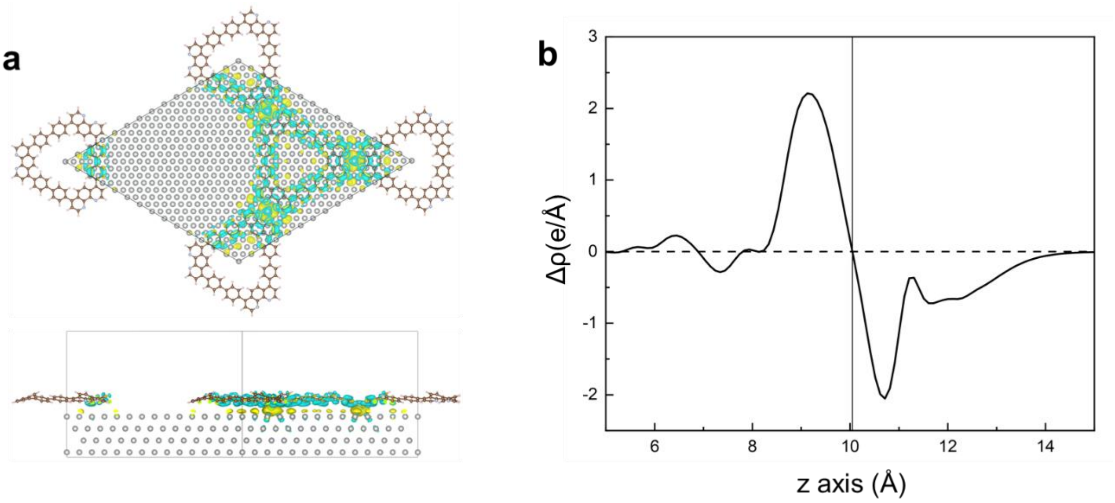

Supplementary Fig. 20 | Calculated Charge density difference. a, Top and side view of the Charge density difference of MOF on Ag(111) surface. **b**, Planar average Charge density difference along z axis. Charge transfer between the MOF structure and Ag(111) substrate is ~2.36 electrons per unit cell.

Supplementary Note 19. The site-dependent Mott gap measurement of the Kagome lattice

The site-dependent gap measurement of the Kagome lattice (Fig. S18). The STS, measured along the linecut 2 by crossing the site of the coordinated Ag, gives a smaller Mott gap of ~85 meV. For other sites of the Kagome lattice, such as the $BPhen_3$ backbone and the $BPhen_3$ center (along the linecut 1), the STS displays a larger Mott gap of ~103 meV.

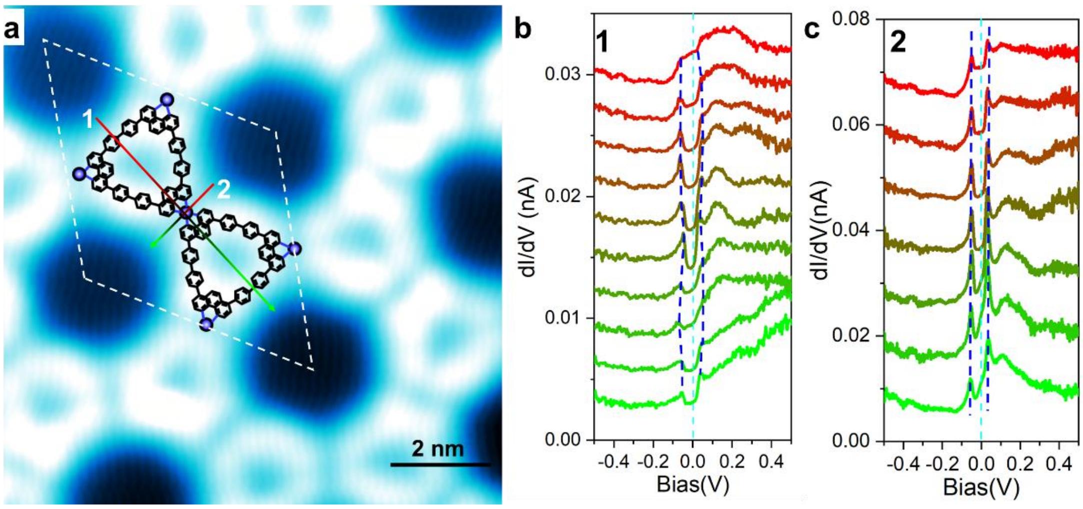

Supplementary Fig. 21 | STS linecuts measured on the framework. a, High-resolution topographic image, measured at V_B= +1400 V and I_T= 300 pA. with two colored arrows indicating the locations of two dI/dV cuts, 1 and 2, respectively. **b**, A line spectroscopic survey taken along the arrowed line 1 in panel **a**. All dI/dV spectra were measured with V_B= -500 mV, I_T= 1000 pA and a bias modulation of 5 mV. **c**, A line spectroscopic survey taken along the arrowed line 2 in panel **a**. All dI/dV spectra were measured with V_B= -500 mV, I_T= 1000 pA and a bias modulation of 5 mV at 4.2 K. The dashed blue lines indicate the evolutions of Mott gap.

Supplementary Note 20. The STS, band structure and dI/dV mapping of the FB2

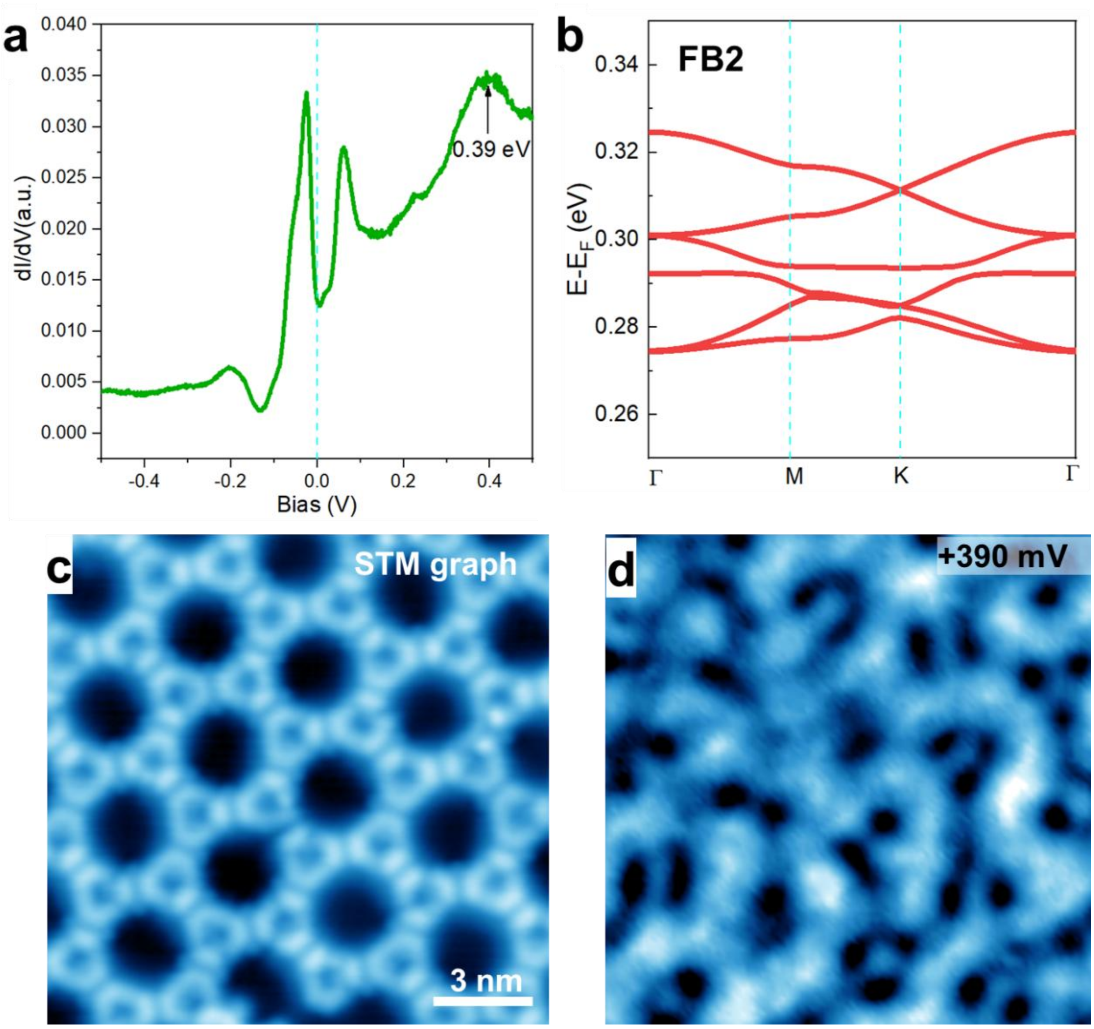

Supplementary Fig. 22 | a, STS measured on the MOF with closer tip-sample distance (U_B=-500 mV; I_T=500 pA) with the bias modulation of 8 mV at 6 K. **b**, the calculated band structure of FB2. **c-d**, topographic image and dI/dV mapping (U_B=+390 mV) taken simultaneously. Image size is 15x15 nm^2.

Supplementary references:

1. Xingyue Wang et al. Impact of Potassium Doping on a Two-Dimensional Kagome Organic Framework on Ag(111), *J. Phys. Chem. Lett.* **16**, 209−214 (2025)

2. Wang, Z. F., Jin, K. H. & Liu, F. Computational design of two dimensional topological materials. *WIREs Comput. Mol. Sci.* **7**, e1304 (2017).

3. Wang, Z. F., Liu, Z. & Liu, F. Organic topological insulators in organometallic lattices. *Nat. Commun.* **4**, 1471 (2013).

4. Wang, Z. F., Liu, Z. & Liu, F. Quantum anomalous Hall effect in 2D organic topological insulators. *Phys. Rev. Lett.* **110**, 196801 (2013).

5. Wang, Z. F., Su, N. & Liu, F. Prediction of a two-dimensional organic topological insulator. *Nano Lett.* **13**, 2842 (2013).

6. Reis, F. *et al.* Bismuthene on a SiC substrate: a candidate for a high-temperature quantum spin Hall material. *Science* **357**, 287 (2017).

7. Tang, S. *et al.* Quantum spin Hall state in monolayer 1T′-WTe_2. *Nat. Phys.* **13**, 683 (2017).

8. Wang, Z. F. *et al.* Topological edge states in a high-temperature superconductor FeSe/SrTiO3(001) film. *Nat. Mater.* **15**, 968 (2016).

9. Bolens, A. & Nagaosa, N. Topological states on the breathing kagome lattice. *Phys. Rev. B* **99**, 165141 (2019).

10. Herrera, M. A. J. *et al.* Corner modes of the breathing kagome lattice: Origin and robustness. *Phys. Rev. B* **105**, 085411 (2022).

11. Kempkes, S. N. *et al.* Robust zero-energy modes in an electronic higher-order topological insulator. *Nat. Mater.* **18**, 1292–1297 (2019).

12. Hu, T., Zhong, W., Zhang, T. *et al.* Identifying topological corner states in two-dimensional metal-organic frameworks. *Nat. Commun.* **14**, 7092 (2023).

13. Jennifer E Hoffman. Spectroscopic scanning tunneling microscopy insights into Fe-based superconductors. *Rep. Prog. Phys.* **74** 124513 (2011)

14. Yin, R., Zhu, X., Fu, Q. *et al.* Artificial kagome lattices of Shockley surface states patterned by halogen hydrogen-bonded organic frameworks. *Nat. Commun.* **15**, 2969 (2024).

15. Requist, R., Sheverdyaeva, P. M., Moras, P., Mahatha, S. K., Carbone, C., and Tosatti, E. Spin-orbit interaction and Dirac cones in d-orbital noble metal surface states. *Phys. Rev. B* **91**, 045432 (2015).
